# Self-Emulsifying Formulation of *Huperzia carinata* Extract to Improve Huperzine A Solubility for Therapeutic Efficacy Enhancement in Parkinsonian Mice

**DOI:** 10.1021/acsomega.6c04075

**Published:** 2026-08-05

**Authors:** Namfa Sermkaew, Nuttapon Songnaka, Wuttipong Masraksa, Amit Jaisi, Yosita Yongyuen, Pannatorn Buntha, Nadiah Syafiqah Nor Azman, Juntratip Jomrit, Phetcharat Boonruamkaew

**Affiliations:** † School of Pharmacy, 65133 Walailak University, Nakhon Si Thammarat 80160, Thailand; ‡ Drug and Cosmetics Excellence Center, Walailak University, Nakhon Si Thammarat 80160, Thailand; § Faculty of Medicine, 155164 Vongchavalitkul University, Nakhon Ratchasima 30000, Thailand; ∥ Faculty of Science, 133942 Chulalongkorn University, Bangkok 10330, Thailand; ⊥ Faculty of Pharmacy, 283706 Lincoln University College, Selangor 47301, Malaysia

## Abstract

Huperzine A (HupA), a sesquiterpene alkaloid isolated from *Huperzia serrata*, is clinically applied in China for Alzheimer’s disease and in the United States as a dietary supplement. In Thailand, *Huperzia carinata* (*H. carinata*) is an alternative and the richest natural source of HupA. However, poor solubility and low oral bioavailability restrict its therapeutic application. This study aimed to develop a self-emulsifying drug delivery system (SEDDS) to improve the solubility, dissolution, and efficacy of HupA from *H. carinata* extract in a mouse model of Parkinson’s disease (PD). The optimized SEDDS consisted of oleic acid (20%), polyethylene glycol-40 hydrogenated castor oil (70%), and propylene glycol (10%), containing 10% *H. carinata* extract. The HupA-loaded SEDDS displayed efficient self-emulsification, producing droplet sizes of 175.2 ± 8.3 nm to 253.4 ± 2.8 nm upon dilution in water. The SEDDS promoted rapid dissolution, achieving approximately 70% HupA release within 5 min and near-complete dissolution (100%) by 120 min in both gastric and intestinal media. *In vitro*, the SEDDS displayed significantly superior antioxidant and anti-inflammatory activities compared with the unformulated extract (*p* < 0.001). *In vivo*, MPTP-induced Parkinsonian mice receiving SEDDS containing HupA (0.75 and 1.00 μmol/kg body weight) showed significant improvements in locomotor function and motor coordination compared with both vehicle- and blank-SEDDS-treated groups (*p* < 0.001) at days 3 and 7 after MPTP induction. Biochemical assessments revealed declined malondialdehyde and nitrite levels in cortical and striatal brain tissues compared with the vehicle- and blank-SEDDS-treated groups (*p* < 0.05). Molecular studies confirmed that HupA-loaded SEDDS increased tyrosine hydroxylase and decreased tumor necrosis factor-α expression compared with the vehicle-treated group (*p* < 0.001) while protecting dopaminergic neurons in immunohistochemistry, indicating neuroprotective and antineuroinflammatory effects. The SEDDS formulation may represent a promising drug delivery system for the clinical application of HupA in PD and potentially other neurodegenerative disorders. This study contributes to Sustainable Development Goal 3 (good health and well-being) by developing neuroprotective formulations for neurodegenerative diseases.

## Graphical abstract

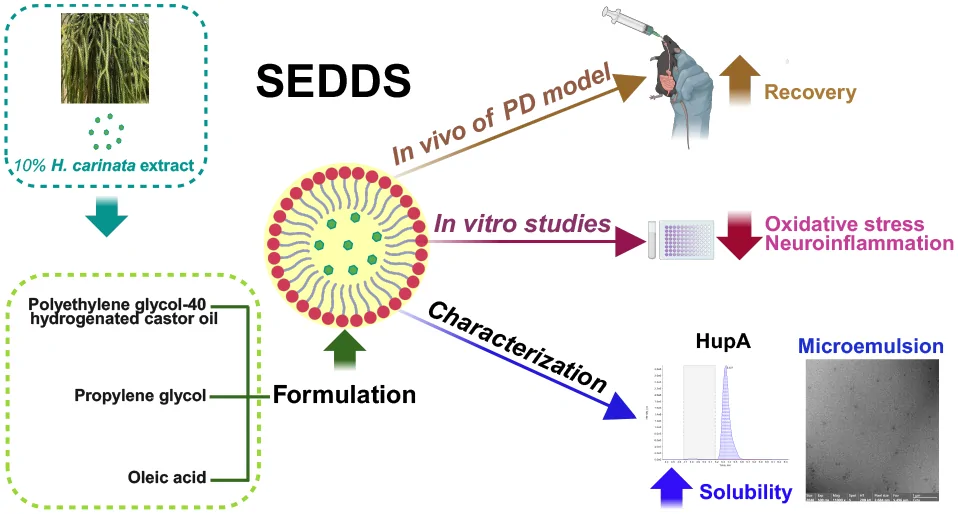

## 1 Introduction

Parkinson’s disease (PD) is a rapidly growing global health concern, particularly among aging populations. In 2021, an estimated 10 million people worldwide were living with PD, representing a 273.76% increase compared with 1990. The projected global burden is expected to exceed 17 million cases by 2035.^1^ The prevalence of PD is particularly high in older adults, with statistics indicating that approximately one in ten individuals over the age of 60 is affected. In Thailand, there were approximately 13.36 million elderly individuals (aged ≥ 60 years), accounting for 19.6% of the total population, with a PD prevalence of 242.57 per 100,000 people.^2^

The rising prevalence of PD globally presents a major public health challenge for healthcare systems, imposing substantial economic and caregiving burdens. This increasing trend underscores the urgent need for disease-modifying therapies and neuroprotective strategies. PD is a progressive neurodegenerative disorder characterized by motor symptoms, such as bradykinesia, resting tremor, rigidity, and postural instability, resulting from the degeneration of dopaminergic neurons in the substantia nigra pars compacta (SNpc). This neuronal loss reduces dopamine levels in the striatum, leading to marked motor dysfunction.^3^ Beyond motor impairment, PD encompasses a broad spectrum of nonmotor symptoms, including cognitive decline, depression, sleep disturbances, and autonomic dysfunction, which substantially reduce quality of life. These manifestations contribute to high levels of disability and caregiving demands as well as considerable stress and burden on caregivers.^4^

The etiology of PD is multifactorial. Exposure to herbicides, pesticides, and the production of reactive oxygen species in the body is mainly related to electron transporter complexes 1 and 2 in the inner mitochondrial membrane. Increased accumulation of reactive oxygen species (ROS) and oxidative stress elicit the reduction of endogenous antioxidant enzymes that can lead to the aggregation of misfolded proteins, DNA damage, autophagy, and apoptosis.^5^ In addition, neuroinflammation may develop in PD. Neuroinflammation is an essential immune response in the central nervous system that maintains neuronal health. In contrast, its neurotoxicity may exacerbate neuronal injury.^6,7^ Currently, an effective approach to alleviate PD pathophysiology is to inhibit oxidative stress and neuroinflammation.

Conventional pharmacological treatments, including levodopa, dopamine agonists, monoamine oxidase-B (MAO-B) inhibitors, and catechol-O-methyltransferase (COMT) inhibitors, are widely used to manage symptoms by replenishing dopamine levels.^2^ However, their long-term use is associated with several side effects and limitations, including motor fluctuations, dyskinesia, and autonomic dysfunction (e.g., nausea and orthostatic hypotension), as well as levodopa-induced neurotoxicity, which can contribute to substantial disability over time.^8^ Moreover, these therapies do not address the underlying neurodegeneration, underscoring the urgent need for alternative treatment approaches that offer both symptomatic relief and neuroprotection. Current research is increasingly focused on natural extracts as potential therapeutic agents to mitigate the adverse effects and limitations of levodopa while also reducing the treatment costs.

Huperzine A (HupA) is a potent natural lycopodium alkaloid chemically identified as (5R,9R)-5-amino-11-ethylidene-7-methyl-5,6,9,10-tetrahydro-5,9-methanocycloocta­[b]­pyridin-2­(1H)-one.^9^ The molecular architecture of HupA is characterized by a rigid tricyclic system fused with a pyridone ring and a primary amine group. Despite the presence of these polar groups, the dominance of the hydrophobic tricyclic framework results in a molecule with limited aqueous solubility. This is reflected in its relatively low Topological Polar Surface Area (TPSA), calculated at approximately 55.1 Å^2^,^10^ which contributes to its classification as a poorly water-soluble compound (Figure 1). HupA was first isolated from *Huperzia serrata* Thunb.) Trevis, a club moss traditionally used in Chinese medicine for its neuroprotective activity.^11^ Its acetylcholinesterase (AChE) inhibitory activity increases acetylcholine levels and improves cognitive function, making it a promising candidate for the treatment of both Alzheimer’s disease and Parkinson’s disease (PD).^12,13^ Beyond AChE inhibition, HupA also exerts potent neuroprotective effects, including antioxidant, anti-inflammatory, and antiapoptotic activities, which may reduce neuronal degeneration in PD.^14,15^

**Figure 1:**
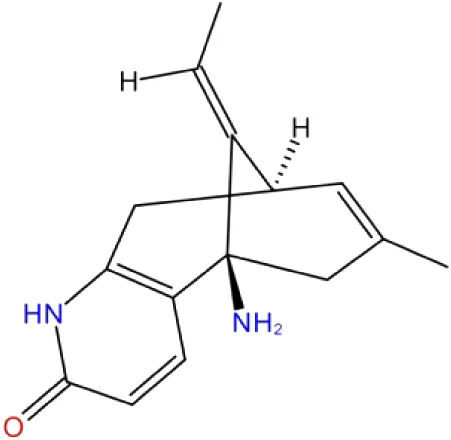
Chemical structure of Huperzine A.

In Thailand, a phytochemical study on the bioactivity and chemical composition of ferns and fern-like plants identified *Huperzia carinata* (Desv.) Trevis (*H. carinata*) as the species with the highest HupA content. Locally known as “Sroi Nang Krong,” *H. carinata* belongs to the Lycopodiaceae family and contains lycopodium alkaloids. However, although HupA is highly soluble in methanol, it is poorly soluble in water.^16^ Its poor aqueous solubility and low oral bioavailability remain major obstacles to clinical application. Therefore, the development of an efficient drug delivery system for HupA is essential to improve its solubility and gastrointestinal absorption.

Self-emulsifying drug delivery systems (SEDDSs) are lipid-based formulations designed to enhance the solubility and oral bioavailability of poorly water-soluble compounds. They are composed of oils, surfactants, and cosolvents, which spontaneously emulsify upon contact with the aqueous environment of the gastrointestinal tract, forming fine oil-in-water (O/W) emulsions with droplet sizes typically ranging from 100 to 300 nm. By promoting solubilization, SEDDSs can improve intestinal permeability and facilitate lymphatic transport, thereby enhancing the systemic bioavailability.^17^ HupA is characterized by poor aqueous solubility and limited oral bioavailability.^16^ Therefore, dissolution is the rate-limiting step for absorption. However, there are no research articles that classify HupA in the Biopharmaceutics Classification System (BCS) class. While conventional approaches such as amorphous solid dispersions and cocrystals are effective solubility enhancement strategies, their application to plant-derived extracts presents practical challenges, including physicochemical instability and conversion of the amorphous drug to a crystalline form (crystallization).^18^ In contrast, SEDDSs are particularly well-suited for delivering lipophilic compounds because they maintain the drug in a solubilized state throughout the gastrointestinal tract, reduce precipitation risk, and improve dissolution reproducibility. Moreover, SEDDSs offer additional advantages by facilitating intestinal absorption through interaction with enterocyte transport pathways, promotion of lymphatic transport, and modulation of the intestinal environment. Moreover, the development and optimization of a SEDDS of *H. carinata* extract have not previously been reported.

*In vivo* studies have confirmed the potential of SEDDSs to improve the bioavailability of poorly soluble compounds. For example, incorporating HupA into a self-microemulsifying drug delivery system (SMEDDS) significantly increased its absorption via the intestinal lymphatic pathway.^16^ Both the area under the curve (AUC) and the maximum plasma concentration (C_max_) of HupA-SMEDDS were significantly higher than those of the HupA suspension, confirming enhanced systemic absorption. Furthermore, the proportion of drugs transported through the lymphatic system was greater in the SMEDDS group than in the suspension group. These findings indicate that SEDDS-based delivery systems represent an effective strategy for improving the oral bioavailability of HupA and other lipophilic drugs. However, the neuroprotective potential of an oral SEDDS of *H. carinata* extract to improve HupA solubility has not previously been investigated through an MPTP-induced Parkinson’s mouse model.

Therefore, this study aimed to develop and optimize a SEDDS formulation of *H. carinata* extract to enhance the solubility and dissolution of HupA. The formulation was characterized physicochemically and further determined for its antioxidant and antineuroinflammatory activities, as well as its neuroprotective efficacy in an MPTP-induced mouse model of PD.

## 2 Results

### 2.1 Composition of *Huperzia carinata* (*H. carinata*) Extract and Fractions by Liquid Chromatography–Tandem Mass Spectrometry (LCMS-MS/MS)

The LCMS-MS/MS analysis of the ethanolic extracts of *H. carinata* showed the presence of 11 Lycopodium alkaloids (Table 1, Supporting Information Figures S1–S11). Huperzine A exhibits more potent neuroprotective effects and selective inhibitory activity against acetylcholinesterase than does Huperzine B.^19,20^ The results indicate that the extract consists of multiple bioactive constituents that may act together to enhance its neuroprotective effect.

**Table 1:** Identification of Lycopodium Alkaloids in the Ethanolic Extracts of *Huperzia carinata* by HPLC/ESI-QTOF-MS

No.	Compound^a^	Retention time (min)	Precursor mass	Monoisotropic mass	Error
1	Huperzine A	3.827	243.1491	242.1410	1.0081
2	Huperzine B	3.421	257.1651	256.1576	1.0075
3	des-*N*-methyl- β-obscurine	2.427	259.1834	258.1732	1.0102
4	Lycopodine	4.281	248.2009	247.1936	1.0073
5	Lycodine	3.141	243.1833	242.1783	1.0050
6	Fawcettimine	4.464	264.1978	263.1885	1.0093
7	Huperzine C	3.627	243.1493	242.1419	1.0059
8	α-obscurine	9.958	275.2759	274.2045	1.0714
9	β-obscurine	2.337	273.1596	272.1889	0.9707
10	Huperzinine	5.624	271.1434	270.1732	0.9707
11	Lycopodine-*N*-oxide	3.648	264.1961	263.1885	1.0076

a Annotation of the compounds is based on fragmentation data.^21^

### 2.2 Development of the Liquid SEDDS of *H. carinata* Extract

#### 2.2.1 Solubility Studies for Excipients Selection

Solubility studies were conducted to assess the solubility of HupA in various oils, surfactants, and cosolvents, with results summarized in Table 2. The solubility of HupA varied considerably among the tested excipients, primarily influenced by polarity, hydrogen-bonding capacity, and the presence of solubilizing agents. HupA, a sesquiterpene alkaloid, contains a polar functional group of lactam, which can interact with the polar groups of excipients. In SEDDS, the oil phase serves as the core component and the primary solubilizing medium for the drug. Among the oils tested, oleic acid exhibited markedly higher solubility for HupA compared to olive oil, soybean oil, and sesame oil. This enhanced solubility is likely due to the free carboxyl group of oleic acid, which can form hydrogen bonds with the polar groups of HupA. In contrast, long-chain triglyceride oils, such as olive, soybean, and sesame oils, demonstrated lower solubility for HupA because of their nonpolar nature and lack of hydrogen-bonding functional groups. Thus, oleic acid showed superior interaction with HupA and proved to be more effective for solubilizing drugs with polar functional groups, such as HupA in *H. carinata* extract.

**Table 2:** Solubility Profile of HupA in *H. carinata* Extract in Different Oils, Surfactants, and Cosolvents^a^ ^c^

Vehicles	Solubility (mg/mL) Mean ± SD
**Oils**	
Oleic acid	0.532 ± 0.167
Olive oil	0.003 ± 0.001^b^
Soybean oil	0.004 ± 0.000^b^
Sesame oil	0.009 ± 0.000^b^
**Surfactants**	
Labrasol	0.047 ± 0.013
PEG-40 hydrogenated castor oil	0.032 ± 0.013
Polysorbate 80	0.029 ± 0.010
**Cosolvents**	
PEG 400	0.058 ± 0.037^c^
Propylene glycol	0.418 ± 0.162

a Data were expressed as mean ± SD, *n* = 5.

b *p* < 0.05 compared to oleic acid.

c *p* < 0.05 compared to propylene glycol.

High hydrophilic–lipophilic balance (HLB) surfactants were selected for the solubility studies due to their ability to form oil-in-water microemulsions upon self-emulsification of the SEDDS in aqueous media. The solubility results showed that Labrasol, polyethylene glycol-40 hydrogenated castor oil, and polysorbate 80 provided comparable solubility for HupA. Consequently, these surfactants were further evaluated in SEDDS formulations based on visual self-emulsification grading, emulsification time, and emulsion droplet size to identify the optimal surfactant that provided the most favorable SEDDS performance.

#### 2.2.2 Formulation of Blank SEDDS

The selection of appropriate excipients is a critical prerequisite for the successful formulation of SEDDS, as only specific combinations of oils, surfactants, and cosolvents result in efficient self-emulsifying systems. Visual grading was used to screen the optimal SEDDS formulations prior to incorporating the *H. carinata* extract. Three systems were prepared based on the solubility study results:

**System A**: Oleic acid/Labrasol/Propylene glycol
**System B**: Oleic acid/PEG-40 hydrogenated castor oil/Propylene glycol
**System C**: Oleic acid/Polysorbate 80/Propylene glycol

Among these, only formulations A1 and B1 exhibited self-emulsifying behavior with a visual grade of B. These two formulations were further evaluated for emulsion droplet size after 50-fold dilution with distilled water, with the goal of identifying formulations that produced droplet sizes within the 50–300 nm range. Formulation A1 produced droplets with a mean size of 307.9 ± 3.0 nm and an emulsification time of 3.38 ± 0.83 min, which exceeded the target size range. In contrast, formulation B1 produced a mean droplet size of 182.7 ± 2.0 nm with a polydispersity index (PDI) of 0.247 ± 0.013, both within the acceptable limits. The emulsification time of B1 was approximately 4.02 ± 0.36 min. Based on these results, formulation B1 was selected for the development of the *H. carinata* extract-loaded SEDDS. The optimal blank formulation (B1) consisted of 20% oleic acid, 70% PEG-40 hydrogenated castor oil, and 10% propylene glycol (Table 3).

**Table 3:** Visual Observation, Emulsification Time, and Mean Droplet Size of Various SEDDS Formulations^a^

		Compositions (%w/w)							
System	Formulation	Oleic acid	Labrasol	PEG-40 hydrogenated castor oil	Polysorbate 80	Propylene glycol	Visual grading	Emulsification time (min) (mean ± SD)	Emulsion droplet size (nm) (mean ± SD)
A	A1	20	70			10	B	3.38 ± 0.83	307.9 ± 3.0
	A2	30	50			20	D	>5	ND
	A3	40	30			30	E	>5	ND
B	B1	20		70		10	B	4.02 ± 0.36	182.7 ± 2.0
	B2	30		50		20	E	>5	ND
	B3	40		30		30	E	>5	ND
C	C1	20			70	10	C	>5	ND
	C2	30			50	20	C	>5	ND
	C3	40			30	30	E	>5	ND

a Data were expressed as mean ± SD, *n* = 3. Note: ND (Not determined).

#### 2.2.3 Physical Characterization of the Optimal SEDDS Formulation Containing *H. carinata* Extract

After incorporation of *H. carinata* extract into the blank B1 SEDDS, the formulation appeared as a dark green solution. The emulsion droplet sizes of the B1 SEDDS containing *H. carinata* extract are presented in Table 4. The robustness to dilution is a critical factor in ensuring the stability and performance of SEDDS under physiological conditions. The results demonstrated that the selected B1 formulation containing *H. carinata* extract maintained mean droplet sizes below 300 nm across all tested dilution ratios. Minimal changes in droplet size were observed at 1:50, 1:100, and 1:500 (v/v) dilutions. However, at a 1:1000 (v/v) dilution, a significant increase in droplet size was observed. This suggests that higher dilution ratios may negatively affect the emulsification process, likely due to reductions in surfactant concentration and increased interfacial tension. Surfactants are crucial for emulsion stabilization, as they reduce the interfacial tension between immiscible phases. An optimal surfactant concentration ensures adequate coverage of the oil–water interface, resulting in smaller and more stable droplets.

**Table 4:** Mean Droplet Size and Polydispersity Index of B1 SEDDS Containing *H. carinata* Extract after Dispersion in Distilled Water at Various Dilution Factors^b^ ^a^

Dilution factors (v/v)	Mean droplet size (nm) ± SD	Polydispersity index (PDI) ± SD
1:50	175.2 ± 8.3	0.257 ± 0.030
1:100	171.2 ± 5.2	0.280 ± 0.040
1:500	187.1 ± 2.9	0.248 ± 0.030
1:1000	253.4 ± 2.8^b^	0.297 ± 0.020

a Data were expressed as mean ± SD, *n* = 3.

b *p* < 0.05 vs dilution factor of 1:50.

#### 2.2.4 Morphology Assessment of Emulsion Droplets Using Transmission Electron Microscopy (TEM)

Microscopic evaluation revealed emulsion droplets ranging in size from 130 to 170 nm, which appeared to be spherical and uniform in shape and size (Figure 2). The observed droplet sizes were consistent with those obtained from the dynamic light scattering measurements.

**Figure 2:**
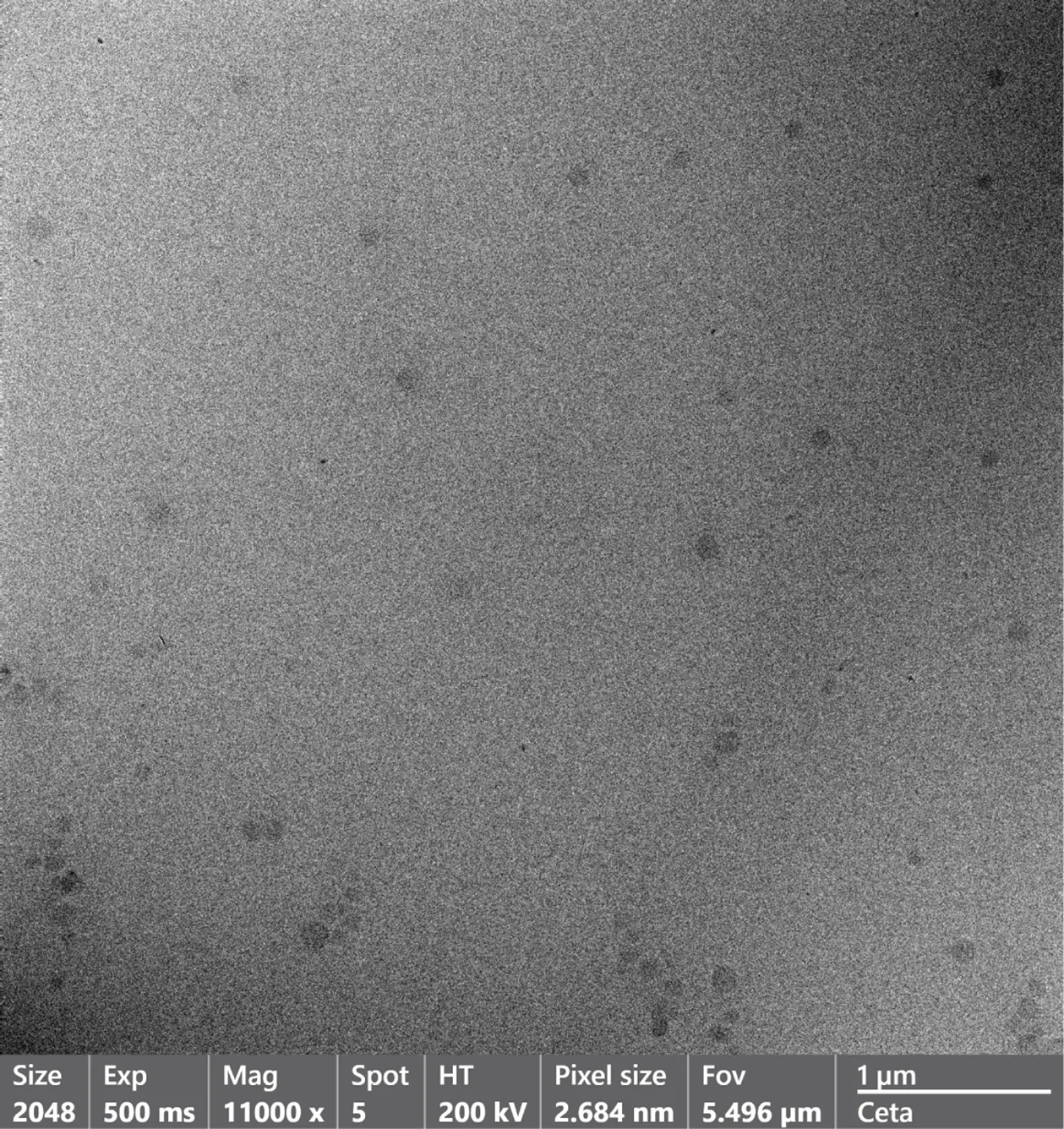
TEM image of B1 SEDDS containing *H. carinata* extract after dilution at 1:50 v/v in distilled water under 11,000× magnification.

#### 2.2.5 *In Vitro* Dissolution Studies

The dissolution profile of the optimized B1 SEDDS formulation containing *H. carinata* extract was compared with that of the unformulated extract in simulated gastric fluid (SGF), pH 1.2 without pepsin, and simulated intestinal fluid (SIF), pH 6.8 without pancreatin. The results are presented as plots of the cumulative percentage of HupA dissolved versus the time (Figure 3).

**Figure 3:**
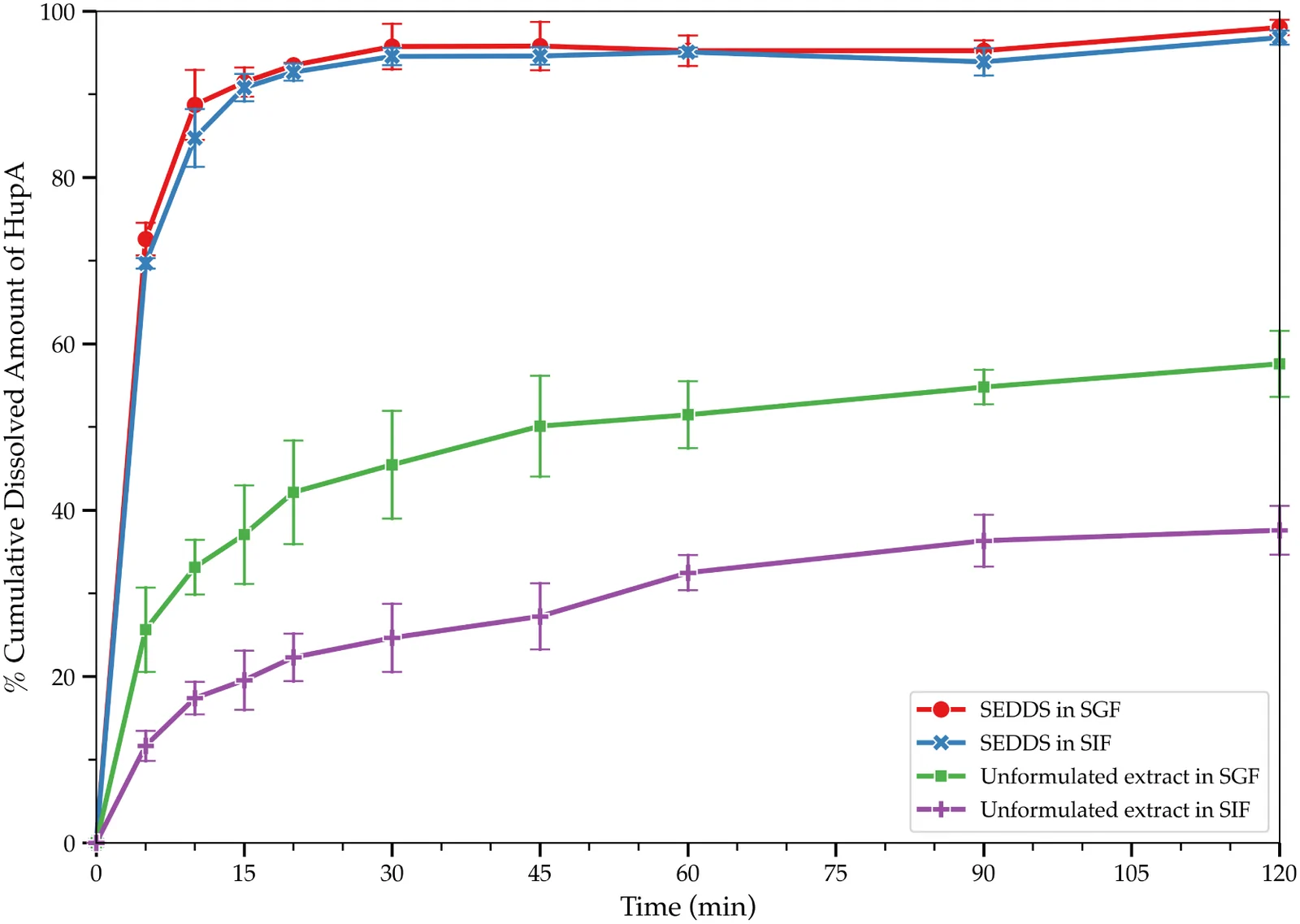
Dissolution profiles of HupA from the optimal SEDDS formulation of *H. carinata* extract (B1) and unformulated *H. carinata* extract in simulated gastric fluid (SGF, pH 1.2) and simulated intestinal fluid (SIF, pH 6.8), respectively. Data were expressed as mean ± SD, *n* = 4.

The SEDDS formulation enhanced solubilization efficiency, addressing the inherent solubility limitations of the unformulated extract. By maintaining the active compound in a solubilized state within the formulation, the SEDDS enabled rapid drug dissolution, with approximately 70% of HupA dissolved within 5 min and near-complete dissolution (∼100%) within 120 min. Upon contact with aqueous media, the SEDDS spontaneously disperses into a fine oil-in-water (O/W) emulsion, effectively removing the requirement for a dissolution step.

This formulation strategy ensured a pH-independent release profile with consistent dissolution rates observed in both simulated gastric fluid (SGF) and simulated intestinal fluid (SIF). In contrast, the unformulated extract reached only 60% in the SGF and 38% in the SIF after 120 min. This disparity is attributed to the alkaloidal nature of HupA, which undergoes protonation in the acidic environment of SGF; the resulting ionized state enhances solubility compared with the relatively un-ionized state in the neutral pH of SIF. Ultimately, the SEDDS formulation enhanced the amount of HupA dissolved by 1.7-fold in SGF and 2.6-fold in SIF compared with the unformulated extract.

### 2.3 *In Vitro* Evaluation of SEDDS Containing *H. carinata* Extract

#### 2.3.1 Antioxidant Properties of SEDDS Containing *H. carinata* Extract Assessed by DPPH Free Radical Scavenging Assay

Evaluation of antioxidant capacity using the DPPH radical scavenging assay revealed an IC_50_ of 29.61 ± 1.536 μg/mL for the SEDDS containing *H. carinata* extract. In comparison, the IC_50_ values were 7.42 ± 2.115 μg/mL for ascorbic acid, 24.10 ± 1.462 μg/mL for α-tocopherol, and 92.68 ± 3.054 μg/mL for the unformulated extract. Thus, the SEDDS demonstrated enhanced antioxidant activity by improving absorption and bioavailability compared with the unformulated extract (*p* < 0.001), although it remained less potent than ascorbic acid (*p* < 0.001). The antioxidant activity of the blank SEDDS was 242.61 ± 1.172 μg/mL. This was significantly different from both the unformulated *H. carinata* extract, ascorbic acid, and α-tocopherol, indicating that the blank SEDDS itself does not possess intrinsic antioxidant effects (Table 5). These findings indicate that the antioxidant activity of *H. carinata* extract was improved when incorporated into the SEDDS formulation.

**Table 5:** Antioxidant Activity of SEDDS Containing *H. carinata* Extract Determined Using the DPPH Radical-Scavenging Assay (Mean ± SD; *n* = 3)

Group	IC_50_ (μg/mL)
SEDDS containing *H. carinata* extract	29.61 ± 1.536^a^ ^b^
Blank-SEDDS	242.61 ± 1.172^a^ ^b^ ^c^
Unformulated *H. carinata* extract	92.68 ± 3.054^b^
Ascorbic acid	7.42 ± 2.115^a^
α-tocopherol	24.10 ± 1.462^a^

a *p* < 0.001 vs unformulated extract.

b *p* < 0.001 vs ascorbic acid.

c *p* < 0.001 vs α-tocopherol.

#### 2.3.2 Anti-Inflammatory Activity of SEDDS Containing *H. carinata* Extract Evaluated Using Albumin Denaturation Assay

In the albumin denaturation inhibition assay, the SEDDS containing *H. carinata* extract demonstrated an IC_50_ of 186.81 ± 1.971 μg/mL. The formulation showed significantly greater inhibition of albumin denaturation than the unformulated extract (348.79 ± 2.340 μg/mL, *p* < 0.001) and the blank SEDDS (829.73 ± 4.655 μg/mL, *p* < 0.001), with no significant difference compared to diclofenac sodium (200.77 ± 1.303 μg/mL), as shown in Table 6.

**Table 6:** Inhibition of Albumin Denaturation by SEDDS Containing *H. carinata* Extract (Mean ± SD; *n* = 3)

Group	IC_50_ (μg/mL)
SEDDS containing *H. carinata* extract	186.81 ± 1.971^a^ ^b^
Blank-SEDDS	829.73 ± 4.655^a^
Unformulated *H. carinata* extract	348.79 ± 2.340
Diclofenac sodium	200.77 ± 1.303^a^ ^b^

a *p* < 0.001 vs unformulated extract.

b *p* < 0.001 vs blank-SEDDS.

### 2.4 SEDDS Containing *H. carinata* Ameliorates Motor Function in Parkinsonian Mice

#### 2.4.1 Effect of SEDDS of *H. carinata* on Locomotor Activity and Motor Coordination

The oral administration of SEDDS did not cause mortality in mice. No substantial alterations were observed in the mean body weight of the treated and control mice. Baseline behavioral assessments prior to MPTP induction in C57BL/6NJcl mice showed no significant differences across the experimental groups, confirming the comparability of their behavioral profiles (data not shown). Mice induced with Parkinsonian symptoms by MPTP exhibited marked impairments in locomotor activity and motor coordination, in contrast to the control mice that received no treatment, thereby confirming the successful establishment of the MPTP-induced Parkinson’s disease model. In the treatment groups, mice administered SEDDS of *H. carinata* extract containing HupA at doses of 0.75 and 1.00 μmol/kg body weight (BW)­exhibited significant improvements in locomotor performance and motor coordination, as assessed by the resting tremor, grid walk, and narrow beam tests, compared with MPTP-induced mice receiving vehicle at 1 day (*p* < 0.05) and at 3 and 7 days (*p* < 0.001) following MPTP induction. The behavioral outcomes were improved when the extract was incorporated into the SEDDS formulation. Interestingly, the groups receiving SEDDS at doses of 0.75 and 1.00 μmol/kg BW did not show significant differences compared with those treated with Stalevo (Figure 4A–C).

**Figure 4:**
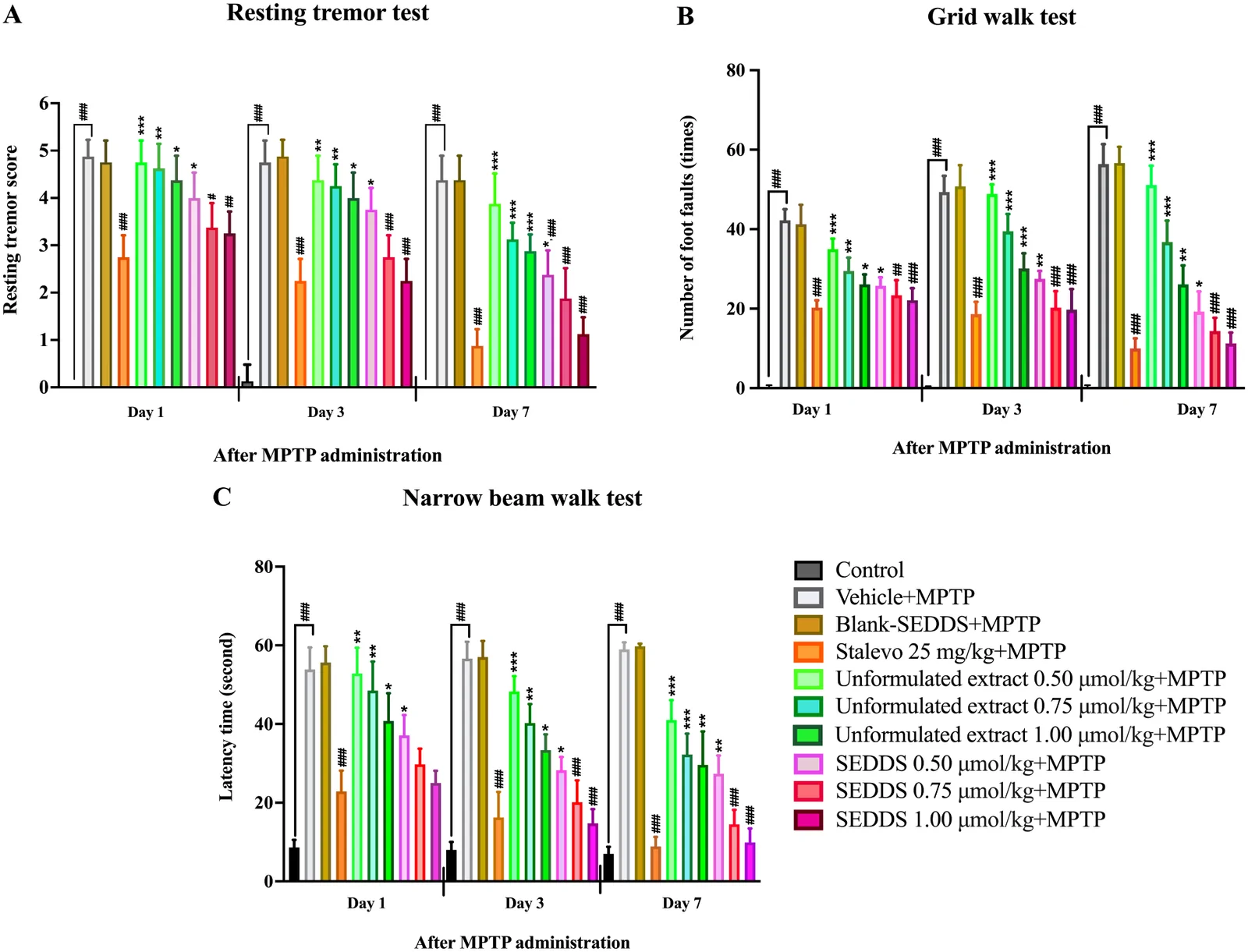
Effect of SEDDS containing *H. carinata* on the resting tremor score of resting tremor test (A), the number of foot faults on grid walk test (B), and the latency time of narrow beam test (C) at 1, 3, and 7 days after MPTP induction. Each data column represents the mean ± SD (*n* = 8/group, ^###^ *p* < 0.001, ^##^ *p* < 0.01, ^#^ *p* < 0.05 vs vehicle + MPTP-treated group, ***p < 0.001, **p < 0.01, *p < 0.05 vs Stalevo + MPTP-treated group).

#### 2.4.2 Antioxidative and Anti-Neuroinflammatory Effects of SEDDS Containing *H. carinata* Extract in MPTP-Induced Parkinsonian Mice

Mechanistic evaluation of the SEDDS formulation containing *H. carinata* extract in MPTP-induced Parkinsonian mice demonstrated marked neuroprotective effects via antioxidative and anti-inflammatory mechanisms. Oral administration of HupA at a dose of 1.00 μmol/kg BW within the SEDDS formulation resulted in a significant reduction of malondialdehyde (MDA) and nitrite levels in both the cortex and striatum compared with the vehicle-treated group (*p* < 0.05). Notably, no significant differences were observed relative to the positive control group. These results indicate that the enhanced bioavailability of HupA provided by the SEDDS formulation contributes to its neuroprotective potential by attenuating oxidative stress and neuroinflammatory processes in the brains of Parkinsonian mice (Figure 5A–B).

**Figure 5:**
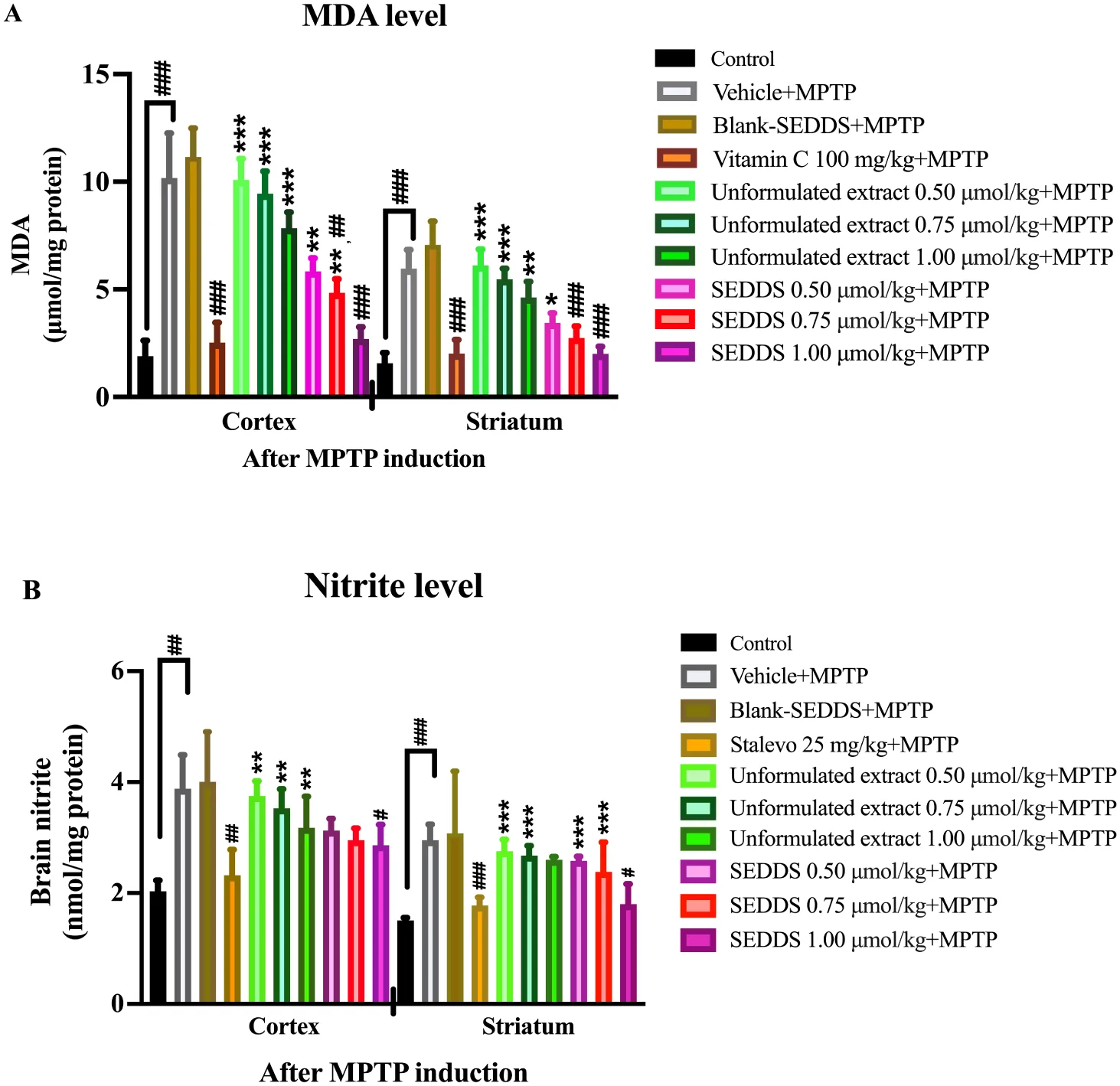
Effect of SEDDS containing *H. carinata* on MDA level (A) and nitrite level (B) after MPTP administration. Each data column represents the mean ± SD (*n* = 8/group, ^###^ *p* < 0.001, ^##^ *p* < 0.01, ^#^ *p* < 0.05 compared with vehicle + MPTP-treated group, ***p < 0.001, **p < 0.01, *p < 0.05 compared with positive control + MPTP-treated group).

#### 2.4.3 Effect of SEDDS of *H. carinata* on TH and TNF-α mRNA Expression in MPTP-Induced Parkinsonian Mice

Quantitative real-time polymerase chain reaction (qPCR) analysis showed that MPTP-induced Parkinsonian mice had significantly reduced TH mRNA expression compared with the control group (*p* < 0.001). Treatment with *H. carinata* extract at 1.00 μmol/kg BW significantly increased TH mRNA expression relative to the vehicle group (*p* < 0.05). Moreover, the self-emulsifying formulation of *H. carinata* extract at 0.50 μmol/kg BW­(*p* < 0.05), 0.75 μmol/kg BW (*p* < 0.001), and 1.00 μmol/kg BW (*p* < 0.001) , as well as Stalevo, significantly upregulated TH mRNA expression compared with the vehicle group (*p* < 0.001) in both the striatum and cortex, as shown in Figure 6A.

**Figure 6:**
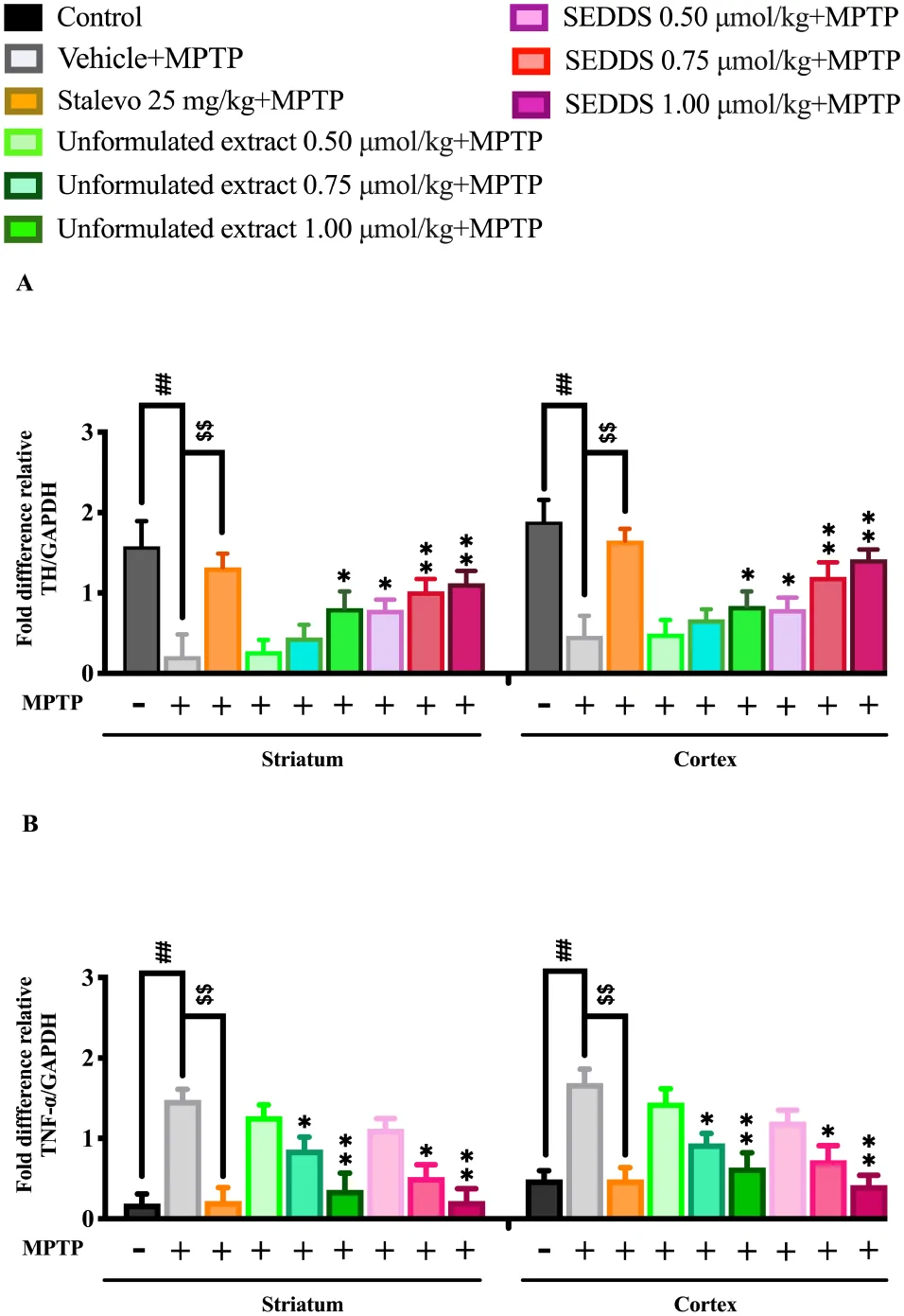
Effect of self-emulsifying formulation of *H. carinata* on TH mRNA (A) and TNF-α mRNA (B) expression in the cerebral cortex and striatum of MPTP-induced parkinsonian mice. Each column represents the mean ± SD (*n* = 5). ^##^ *p* < 0.001 vs the control group; *p < 0.05, **p < 0.001 vs the vehicle-treated group + MPTP; and ^$$^ *p* < 0.001 vs the Stalevo-treated group + MPTP.

In contrast, MPTP-induced Parkinsonian mice exhibited significantly increased TNF-α mRNA expression compared with the control group (*p* < 0.001). Treatment with *H. carinata* extract at 1.00 μmol/kg BW significantly reduced TNF-α mRNA expression relative to the vehicle group (*p* < 0.05), with stronger effects observed at 0.75 μmol/kg BW (*p* < 0.05) and 1.00 μmol/kg BW (*p* < 0.001) . Furthermore, the self-emulsifying formulation of *H. carinata* extract at 0.75 μmol/kg BW (*p* < 0.05) and 1.00 μmol/kg BW­(*p* < 0.001) , as well as Stalevo, significantly decreased TNF-α mRNA expression compared with the vehicle group (*p* < 0.001) in both the striatum and cortex, as shown in Figure 6B.

#### 2.4.4 Treatment with *H. carinata* SEDDS Increased TH-Positive Neurons in MPTP-Induced Parkinsonian Mice

Tyrosine hydroxylase (TH) is the rate-limiting enzyme in dopamine (DA) biosynthesis within dopaminergic neurons. In this study, we evaluated the TH-positive cells in mouse brain sections, particularly within the substantia nigra pars compacta (SNpc). Representative micrographs of TH immunostaining revealed abundant TH-positive dopaminergic neurons in the control group (Figure 7A). In contrast, vehicle-treated and blank-SEDDS mice subjected to MPTP administration showed a marked depletion of TH-immunopositive cells (Figure 7B–C), whereas treatment with Stalevo + MPTP markedly restored TH-immunopositive cells (Figure 7D). Notably, treatment with the SEDDS containing *H. carinata* extract at doses of 0.75 and 1.00 μmol/kg BW significantly ameliorated the loss of TH-immunopositive cells in the SNpc of MPTP-treated mice (Figure 7I–J) compared with the unformulated *H. carinata* extract at doses ranging from 0.50–1.00 μmol/kg BW (Figure 7E–G).

**Figure 7:**
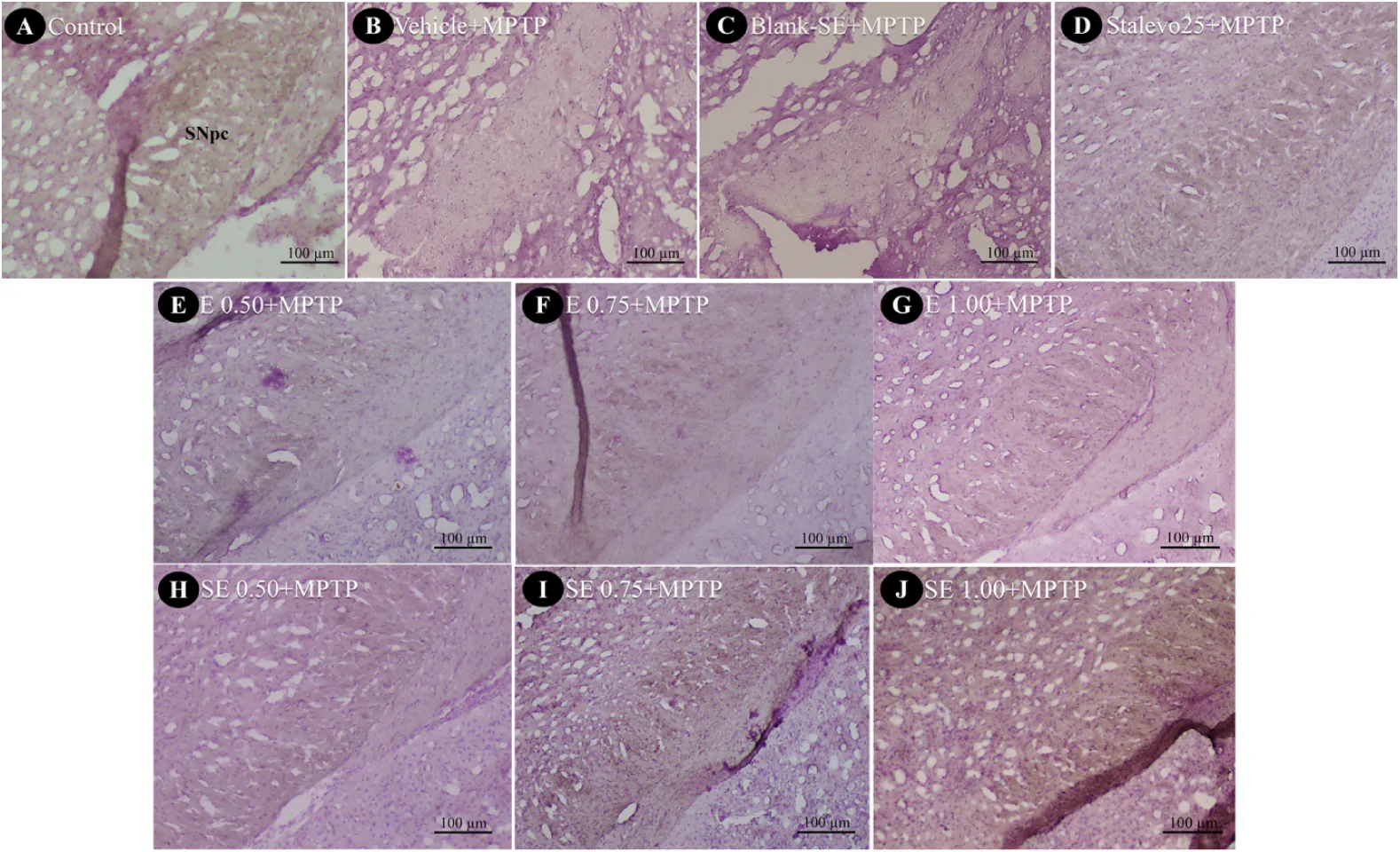
Representative microphotographs showing the effect of SEDDS containing *H. carinata* on TH immunostaining in the SNpc of MPTP-treated mice, counterstained with hematoxylin. Panel A: control group; Panel B: vehicle + MPTP; Panel C: blank-SEDDS + MPTP; Panel D: Stalevo (25 mg/kg BW) + MPTP; Panels E–G: unformulated *H. carinata* extract (0.50, 0.75, and 1.00 μmol/kg BW) + MPTP; and Panels H–J: SEDDS (0.50, 0.75, and 1.00 μmol/kg BW) + MPTP.

## 3 Discussion

Levodopa continues to be the first-line and most effective oral treatment for controlling the symptoms of Parkinson’s disease. Nonetheless, prolonged administration is linked to significant adverse effects, including motor fluctuations, dyskinesias, autonomic disturbances (such as nausea and orthostatic hypotension), and potential levodopa-induced neurotoxicity, all of which can contribute to progressive disability over time.^8^ Consequently, alternative therapeutic approaches have been explored.

Oral administration is considered a safe, convenient, and noninvasive route. For absorption into systemic circulation, drugs must first dissolve in gastrointestinal (GI) fluids.^22^ However, over 40% of newly developed drug molecules exhibit poor water solubility, resulting in limited absorption and low bioavailability.^23–25^ Similarly, HupA, a bioactive alkaloid derived from *H. carinata* with strong neuroprotective potential in Alzheimer’s disease (AD), circumvents these inherent limitations. To address this issue, various strategies have been investigated, among which SEDDS have recently gained increasing attention as promising carriers for enhancing drug delivery.^26^

The Biopharmaceutics Classification System (BCS) classifies drugs according to their solubility and permeability. SEDDS are particularly useful for improving the oral absorption of poorly water-soluble compounds by enhancing their solubility. Solubility screening showed that HupA exhibited different solubilities among the tested excipients, as summarized in Table 2. Among the oil phases, oleic acid provided the highest solubility for HupA, likely due to hydrogen-bonding interactions between the polar functional groups of HupA and the carboxyl group of oleic acid. In contrast, triglyceride-based oils showed lower solubilizing capacity because of their predominantly nonpolar nature. Therefore, oleic acid was selected as the oil phase for the SEDDS formulation.^27^ Oleic acid, being an amphiphilic compound with inherent surfactant properties, is increasingly used as a substitute for conventional triglyceride oils in SEDDS formulations.^28^ Surfactants suitable for SEDDS formulations should exhibit relatively high hydrophilic–lipophilic balance (HLB) values and strong hydrophilicity to promote the rapid formation of O/W droplets upon contact with aqueous media.^17^ Among the surfactants evaluated, Labrasol, PEG-40 hydrogenated castor oil, and Polysorbate 80 exhibited comparable solubilizing abilities, likely due to their similar amphiphilic properties and HLB values. These surfactants also possess similar HLB values: PEG-40 hydrogenated castor oil (HLB 14.3),^29^ Labrasol (HLB 14),^30^ and Polysorbate 80 (HLB 15).^31^ Propylene glycol provided significantly higher HupA solubility than PEG 400 and was selected as the cosolvent. The enhanced solubility is likely due to the solvent’s ability to form hydrogen bonds with polar functional groups in HupA.^32^ Based on these findings, oleic acid, PEG-40 hydrogenated castor oil, and propylene glycol were chosen for the development of the SEDDS formulation to enhance the solubility and potential oral bioavailability of HupA.

Physical characterization of the optimal SEDDS formulation containing *H. carinata* extract is presented in Table 3. The optimized B1 formulation, containing *H. carinata* extract, consistently maintained mean droplet sizes below 300 nm across all tested dilution ratios. The polydispersity index (PDI) is an important parameter for evaluating droplet size uniformity. PDI values below 0.3 generally indicate a monodisperse system with high uniformity.^33^ In this study, the PDI values of the B1 formulation containing *H. carinata* extract ranged from 0.248 to 0.297 across dilution factors from 1:50 to 1:1000, indicating a homogeneous droplet population after dispersion (Table 4). Overall, these results suggest that while the B1 formulation containing *H. carinata* extract exhibits robustness to dilution, especially at lower dilution levels, the PDI values confirm a uniform and stable emulsion system across the tested ranges.

Microscopic evaluation of TEM showed uniform spherical emulsion droplets (130–170 nm), indicating a small droplet size favorable for oral drug absorption (Figure 2). The small droplet size is an important feature, as it can enhance drug absorption into the systemic circulation following oral administration.

SEDDS are promising delivery systems that facilitate the formation of O/W emulsions in the gastrointestinal (GI) tract. These formulations maintain the drug in a solubilized state, and the increased surface area resulting from the nanosized droplets can improve membrane permeability, promote lymphatic transport, and ultimately enhance oral bioavailability.^34^ In addition, SEDDS formulations promote wide drug distribution within the stomach and GI tract and lessen irritation from extended intestinal wall contact.^17^

As a result of the dissolution profile of the optimized B1 SEDDS formulation containing *H. carinata* extract (Figure 3), HupA was released rapidly without the need for an additional solubilization step. Aqueous solubility and the dissolution rate are critical prerequisites for oral absorption. Upon dispersion in aqueous media such as gastrointestinal fluid, SEDDSs formed a fine O/W emulsion with a large surface area, facilitating rapid drug release and promoting efficient absorption.^35^ The observed variability may be partly attributed to the complex composition of the unformulated *H. carinata* extract, which contains multiple constituents with different physicochemical properties. Differences in the dispersion of the crude extract in SGF and SIF during the dissolution test may also contribute to the variability among replicates.

The distinctive feature of the present formulation lies not in the distinct excipients but in their specific composition and proportions, which were optimized for the *H. carinata* extract. The optimized SEDDS contained oleic acid, PEG-40 hydrogenated castor oil, and propylene glycol, incorporated 10% (w/w) extract, formed droplets smaller than 300 nm, and improved HupA dissolution by approximately 1.7-fold in SGF and 2.6-fold in SIF compared with the unformulated extract. Unlike systems developed for purified HupA or conventional extract preparations, this formulation was designed to disperse a chemically complex plant extract while retaining other constituents that may contribute to its biological activity.

Increased accumulation of ROS and oxidative stress triggers the reduction of endogenous antioxidant enzymes that can lead to the aggregation of misfolded proteins, DNA damage, autophagy, and apoptosis.^5^ The *in vitro* antioxidant activity of SEDDS containing *H. carinata* extract was significantly higher than that of the unformulated crude extract (*p* < 0.001), indicating enhanced bioefficacy of HupA following formulation (Table 5). Our results concur with previous evidence demonstrating that the piperine self-nanoemulsifying drug delivery system demonstrated maximal scavenging activity at 250 μg/mL, which was significantly higher (*p* < 0.001) than that of the piperine extract dispersion.^36^ In addition, the anti-inflammatory effect of SEDDS containing *H. carinata* extract showed significantly greater inhibition of albumin denaturation compared with the unformulated crude extract (*p* < 0.001). Interestingly, no significant difference was observed when comparing SEDDS containing *H. carinata* extract with diclofenac sodium (Table 6). Our results concur with previous evidence demonstrating that azithromycin incorporated into a chitosan- and Tween 20-based O/W macroemulsion demonstrated concentration-dependent anti-inflammatory activities (8, 16, 32, and 40 μg/mL) in the inhibition of albumin denaturation assay.^37^

Administration of SEDDS by the oral route for 28 days caused no mortality in mice, and the mean body weight of treated animals remained comparable to that of the controls (data not shown). Mice treated with the SEDDS formulation of *H. carinata* extract containing HupA at doses of 0.75 and 1.00 μmol/kg BW demonstrated significant enhancements in locomotor activity and motor coordination, as evidenced by improvements in resting tremor, grid walk, and narrow beam performance, compared with MPTP-induced mice receiving vehicle at day 1 (*p* < 0.05) and at days 3 and 7 (*p* < 0.001) post-MPTP administration (Figure 4). The behavioral enhancements were markedly greater when the extract was administered through the SEDDS formulation. The therapeutic efficacy in a mouse model of PD was observed at remarkably low doses and is attributed to the high potency of Huperzine A and the enhanced bioavailability provided by the SEDDS. Based on the HPLC quantification of the extract, the administered doses correspond to a HupA intake of 0.5, 0.75, and 1.0 μmol/kg BW. Consistent with our findings, a previous study showed that CoQ10 nanoemulsion significantly enhanced performance in locomotor activity, muscle coordination, and akinesia tests compared with CoQ10 suspension by mitigating nigrostriatal dopamine depletion and thereby contributing to the management of Parkinson’s disease.^38^

Excessive production of nitric oxide (NO) has been strongly implicated in neuroinflammation and oxidative/nitrosative stress, contributing to the progression of neuronal damage in Parkinson’s disease.^39^ Accordingly, malondialdehyde (MDA) and nitrite levels were quantified (Figure 5). Biochemical analysis revealed that oral administration of SEDDS containing *H. carinata* (1.00 μmol/kg BW) significantly reduced cortical and striatal MDA and nitrite levels compared with the vehicle-treated group (*p* < 0.05), with effects comparable to the positive control. These findings are in line with previous studies demonstrating that pretreatment with carvacrol, a monoterpenic phenol found in *Nigella sativa*, significantly decreased MDA and nitrite levels while concurrently enhancing catalase activity in the midbrain of a 6-OHDA-lesioned rat model of Parkinson’s disease.^40^

TNF-α is a pivotal pleiotropic cytokine that regulates both neuronal survival and apoptotic death. It has been shown to stimulate mitochondrial reactive oxygen species generation,^41^ potentially compromising the survival of dopaminergic neurons, in which tyrosine hydroxylase (TH) serves as the rate-limiting enzyme, thereby contributing to the pathogenesis of Parkinson’s disease. The molecular findings demonstrate that the SEDDS of *H. carinata* extract provides enhanced neuroprotective and antineuroinflammatory effects in the MPTP-induced Parkinson’s disease model, as illustrated in Figure 6. The SEDDS containing *H. carinata* extract at doses of 0.75 μmol/kg BW (*p* < 0.05) and 1.00 μmol/kg BW (*p* < 0.001) significantly reduced TNF-α mRNA expression while enhancing TH mRNA expression compared with the vehicle group (*p* < 0.001) in both the striatum and cortex of MPTP-induced parkinsonian mice. The therapeutic benefits are mediated through dual mechanisms: restoration of dopaminergic function via TH upregulation and suppression of neuroinflammation through TNF-α downregulation. The improved bioavailability of the self-emulsifying formulation appears to enhance therapeutic efficacy compared to the unformulated extract. Previous literature similarly reported that administration of the α-pinene self-emulsifying nanoformulation enhanced the antioxidant defense system and protected SH-SY5Y cells and the rat PD model against 6-hydroxydopamine more than the α-pinene suspension.^42^

Studies have shown that HupA reduces the levels of various pro-inflammatory cytokines, including iNOS, IL-1β, IL-18, CD16, and TNF-α.^43^ Additionally, HupA injection restores the transcriptional levels of inflammatory factors in the SNpc and striatum tissues of Parkinsonian mice.^15^ In addition, HupA diminishes oxidative brain damage by increasing endogenous radical scavenging enzymes (e.g., superoxide dismutase, catalase, and glutathione peroxidase) and directly scavenging free radicals. These effects help prevent neuronal lipids, proteins, and DNA damage, thereby preserving neuronal integrity under conditions such as traumatic brain injury, neurodegenerative diseases, and stroke.^44^ The dissolving microneedles for transdermal delivery of HupA substantially ameliorate cognitive function in Alzheimer’s disease (AD) rats treated with dissolving microneedle patch-HupA and oral gavage-HupA.^45^ Microemulsion-based patch for transdermal delivery of HupA and ligustrazine phosphate significantly improved memory in scopolamine-induced amnesia rats.^46^ Poly­(3-hydroxybutyrate-co-3-hydroxyvalerate-co-3-hydroxyhexanoate)-based microspheres for Huperzine A improved function in AD mice with slower HupA release and lower toxicity.^47^

Our immunohistological analysis (Figure 7) revealed a pronounced reduction of TH immunoreactivity in the SNpc 7 days after acute MPTP induction (*i.p.*) in the vehicle- and blank-SEDDS-treated groups (Figure 7B–C), compared with the control group that did not receive MPTP (Figure 7A). Treatment with the SEDDS formulation at doses of 0.75–1.00 μmol/kg BW in the mouse model of PD progressively restored the abundance of TH-positive cells (Figure 7I–J) relative to the vehicle + MPTP and blank-SEDDS + MPTP-treated groups.

HupA is a poorly water-soluble compound, and its effective oral absorption requires adequate solubilization in the gastrointestinal tract. SEDDS form fine emulsion droplets in which the drug is encapsulated within the lipid phase, thereby overcoming the major limitation of low oral bioavailability associated with poor aqueous solubility or permeability. Lipid-based formulations represent an effective strategy for enhancing the solubility and oral bioavailability of lipophilic drugs by maintaining them in a dissolved state after oral administration. Upon exposure to gastrointestinal fluids, SEDDS rapidly disintegrate and self-emulsify under gentle gastric agitation. The lipid components enhance drug absorption through multiple mechanisms, including interaction with enterocyte transport pathways, promotion of lymphatic transport, and modulation of the intestinal environment. Furthermore, gastrointestinal dispersion, lipid digestion, and the solubilization capacity of the formulation collectively govern drug absorption from SEDDSs.^27^

The enhanced solubility and dissolution of HupA may increase its availability for gastrointestinal absorption and partly contribute to improved motor performance, reduced oxidative stress and inflammatory markers, and restored TH expression. However, pharmacokinetic parameters and HupA concentrations in the plasma and brain tissue of the experimental animals were not determined, which represents a limitation of this study.

This study demonstrates the enhanced efficacy of *H. carinata* extract when delivered via SEDDS. The use of a whole extract introduces a complex alkaloid matrix. While HupA is the primary bioactive marker, other minor alkaloids (e.g., Huperzine B (HupB)) may contribute to the observed neuroprotective and anti-inflammatory activities. These pharmacological activities are directly relevant to the pathophysiological processes involved in Parkinson’s disease. The potential for synergistic effects versus the activity of pure HupA will be addressed in future comparative studies to fully clarify the multicomponent contributions. HupA exhibits more potent neuroprotective effects and selective inhibitory activity against acetylcholinesterase than Huperzine B.^19,20^ PC12 cells pretreated with Huperzine B before H_2_O_2_ induction notably enhanced the cell survival and radical scavenging enzyme activities and reduced the lipid peroxidation.^20^ HupB exhibits a higher therapeutic index than HupA and possesses other pharmacological effects.^48–52^ On the other hand, the synergistic effect of caffeic acid and ferulic acid strengthens the HupA-regulated neuroprotective effect for neurons and the neurite network from glutamate injury.^13^ Pretreatment with HupA and astaxanthin reduced oxidative stress and neurotoxicity induced by β-amyloid, Aβ25–35.^53^

Recent studies have confirmed the neuroactive potential of HupA in neurodegenerative disorders through both *in vitro* and *in vivo* investigations,^19^ findings that are consistent with our results. Based on these findings, the improved solubility and dissolution of HupA achieved through the SEDDS formulation may have enhanced its availability after oral administration, which could partly explain the neuroprotective effects observed in the MPTP-induced Parkinsonian mouse model.

## 4 Conclusion

The development of SEDDS for *H. carinata* extract may enhance the solubility of HupA, a neuroprotective agent with potential benefits in the treatment of Parkinson’s disease. The optimized formulation (B1: 20% oleic acid, 70% PEG-40 hydrogenated castor oil, and 10% propylene glycol containing 10% *H. carinata* extract) exhibited effective self-emulsification and demonstrated enhanced *in vitro* drug dissolution, antioxidant and anti-inflammatory activities, as well as neuroprotective effects in an MPTP-induced Parkinsonian mouse model. These findings highlight the feasibility of this drug delivery approach for the management of Parkinson’s disease and related neurological disorders. However, further pharmacokinetic and toxicity studies are required to confirm its clinical potential.

## 5 Materials and Methods

### 5.1 Materials

Oleic acid, Polysorbate 80, PEG-40 hydrogenated castor oil, polyethylene glycol 400, and propylene glycol were purchased from P.C. Drug Center Ltd., Bangkok, Thailand. Labrasol (Caprylocaproyl Polyoxyl-8 glycerides) was purchased from Gattefossé, Paris, France. Methanol (HPLC grade), acetonitrile (HPLC grade), and hydrochloric acid were purchased from RCI Labscan Ltd., Bangkok, Thailand. Formic acid was purchased from Merck KGaA, Darmstadt, Germany. Huperzine A and MPTP were purchased from Merck Ltd., Bangkok, Thailand. All other chemicals were of analytical reagent grade.

### 5.2 Plant Materials and Extraction

*Huperzia carinata* (Desv.) Trevis, or *H. carinata*, was purchased from the Potsadet garden, Nakhon Si Thammarat. The plant was maintained at the greenhouse at Walailak Botanical Park, and a voucher specimen (WUNST072024HC) was deposited at the herbarium of the Walailak Botanical Park. The collected extracts were further freeze-dried to remove water and kept at −20 °C until further use. The aerial part of the plants was washed in tap water and oven-dried for 36 h at 50 °C. The dried plant material was crushed into fine powder using a grinder and passed through a no. 80 sieve. The extraction uses the method with some modification.^54^ Briefly, 500 g of dried powdered plant material was extracted by 1,500 mL of 80% ethanol (w/v, 1:3) and placed in an ultrasonic bath for 20 min at 25 °C. This was followed by Microwave Assisted Extraction (MAE) at a power of 360 W with three irradiation cycles (each cycle: 2 min power-on and 1 min power-off) and three extraction times. Finally, the pooled extracts were evaporated to dryness by a rotary evaporator at 45 °C and further freeze-dried. The yield of the extract was 59.55 g and was kept at −20 °C until further use. The *H. carinata* extract was quantitatively standardized for its Huperzine A content via HPLC analysis with UV detection at 308 nm. Samples were injected at a volume of 10 μL and eluted at a flow rate of 0.6 mL/min using a mobile phase consisting of acetonitrile (A) and 0.1% formic acid (B). HupA standard solutions were freshly prepared before analysis. The concentration of Huperzine A in the crude extract was found to be 0.52 ± 0.04% (w/w). This standardized extract was subsequently used for all formulation development and biological evaluations to ensure dosage consistency.

### 5.3 Liquid Chromatography–Tandem Mass Spectrometry (LCMS-MS/MS)

LCMS was used for qualitative analysis. It was carried out using High-Performance Liquid Chromatography (HPLC) Thermo Dionex Ultimate 3000 with a SCIEX TripleTOF 6600+ mass spectrophotometer. The metabolites were separated on a reverse-phase column (Acclaim RSLC 120 C18) following a gradient between water containing 0.1% formic acid (A) and acetonitrile with 0.1% formic acid (B): 0–2 min, 5% B; 2–20 min, 5–95% B; 20–25 min, 95% B; 25.5–30 min, 95–5% B. The flow rate was kept at 0.3 mL min^–1^. The mass spectra were acquired by electrospray ionization (ESI) in the negative and positive modes, and a mass range of *m*/*z* 100–1,500 Da was used for the mass spectral analysis with intensity exceeding 100 cps for 25 compounds per cycle for MS2 50–1500 Da (collision energy 40).

### 5.4 Development of the Liquid SEDDS of *H. carinata* Extract

#### 5.4.1 Solubility Studies

The solubility of an *H. carinata* extract in various oils, surfactants, and cosolvents was determined. A 200 mg portion of the extract was added to each microtube containing 1 mL of each vehicle (*n* = 5). Then, mixtures were vortexed (Vortex-genie 2, Becthai Bangkok Equipment & Chemical Ltd., Bangkok, Thailand) and then shaken in an incubator shaker (New Brunswick Innova 44/44R, Eppendorf Ltd., Hamburg, Germany) at 25 °C for 72 h. After incubation, the samples were centrifuged (Centrifuge 5810 R, Eppendorf, Germany) at 6000 rpm for 10 min at 30 °C. The supernatants were appropriately diluted with methanol and filtered through a 0.45 μm membrane filter.^55^ The concentrations of HupA in the supernatant were determined by HPLC. The vehicles that yielded the highest HupA solubility were selected for further formulation development.

#### 5.4.2 Formulation of SEDDS

The compositions that showed high HupA solubility from the solubility tests were weighed into 20 mL glass vials and mixed using a vortex mixer. The concentration range of each component was as follows: 10–50% oil, 25–70% surfactant, and 0–30% cosolvent. The self-emulsifying properties of the SEDDS formulations were evaluated visually. One milliliter of each formulation was dispersed into 50 mL of distilled water (1:50 v/v) in a glass beaker at 25 °C, and the content was gently stirred using a magnetic stir bar. The efficiency of self-emulsification was assessed based on visual observation and scored using a grading system^56^ as follows:

**Grade A**: Clear nanoemulsion that forms quickly and spontaneously.
**Grade B**: Bluish, slightly less clear nanoemulsion that forms quickly and spontaneously.
**Grade C**: Turbid emulsion that forms slowly.
**Grade D**: Turbid emulsion that is dull and grayish.
**Grade E**: Turbid emulsion with visible oil globules on the surface.

In this experiment, formulations that achieved grade A or B and dispersed easily in water within 5 min were selected to determine the mean emulsion droplet size. The selected SEDDS formulations that produced emulsion droplet sizes ranging from 50 to 300 nm upon aqueous dilution were chosen for the development of the *H. carinata* extract-loaded SEDDS.

#### 5.4.3 Preparation of SEDDS Containing *H. carinata* Extract

SEDDS formulations with desirable component ratios were homogenized using an ULTRA-TURRAX homogenizer. The formulations were homogenized at a rotational speed of 3800 rpm at 25 °C. Each batch consisted of 30 g of formulation containing 3 g of *H. carinata* extract (10% w/w of the extract). The mixture was continuously homogenized until a clear solution was obtained. The resulting liquid SEDDS was then manually filled into hard gelatin capsules (size 00), with each capsule containing approximately 950 mg of the formulation.

#### 5.4.4 Determination of Emulsion Droplet Size

The mean droplet size and polydispersity index (PDI) of the emulsion globules were determined using a Zetasizer (Malvern Instruments Ltd., Malvern, UK) based on dynamic light scattering. The SEDDS formulations were diluted with distilled water at a ratio of 1:50 (v/v) and gently stirred with a magnetic stirrer for 5 min before the measurement. Each measurement was performed with 15 subruns per sample. To evaluate the effect of the dilution volume on the physical characteristics of the SEDDS, *H. carinata* extract-loaded formulations were diluted in distilled water at various ratios (1:50, 1:100, 1:500, and 1:1000 v/v). The diluted samples were gently stirred for 5 min by using a magnetic stirrer prior to analysis. The effects of dilution on the emulsion droplet size were then assessed.

#### 5.4.5 *In Vitro* Dissolution Studies

Dissolution studies of the optimized SEDDS formulation were conducted and compared with the unformulated *H. carinata* extract, both loaded into hard gelatin capsules containing an equivalent amount of HupA. The study was carried out using the USP Dissolution Apparatus I with 500 mL of simulated gastric fluid (SGF, pH 1.2) without pepsin or simulated intestinal fluid (SIF, pH 6.8) without pancreatin at 37.0 ± 0.5 °C and a basket rotation speed of 100 rpm. The formulations were subjected to dissolution testing over a 2-h period. At predetermined time intervals (5, 10, 15, 20, 30, 45, 60, 90, and 120 min), 2 mL of the sample was withdrawn and immediately replaced with an equal volume of fresh SGF or SIF. Samples were filtered through a 0.45 μm membrane filter, and the concentrations of HupA were analyzed by HPLC using the analytical method described in Section 5.2. Each test was performed in quadruplicate. The cumulative percentage of HupA dissolved over time was plotted to construct the drug dissolution profiles.

### 5.5 *In Vitro* Study of Antioxidant and Anti-Inflammatory Activities of SEDDS Containing *H. carinata* Extract

#### 5.5.1 Inhibition of 2,2-Diphenyl-1-Picrylhydrazyl (DPPH) Assay

The antioxidant potential of the SEDDS formulated with *H. carinata* extract was examined following the procedure.^57^ A 200 μL portion of either the sample or ascorbic acid (Gammaco Ltd., Bangkok, Thailand) was mixed with 2 mL of a 0.1 mM DPPH solution (Merck Ltd., Bangkok, Thailand) and incubated at 25 °C in the dark for 30 min. The reduction of DPPH, reflected by a color shift from purple to pale yellow, was quantified at 517 nm using a UV–visible spectrophotometer. The free radical scavenging capacity of the SEDDS containing *H. carinata* extract was determined from three independent replicates and reported as IC_50_ values, calculated using the equation below:

$$\mathrm{Inhibition of DPPH}\left(\%\right)=\left[\left({\mathrm{Absorbance}}_{\mathrm{control}}-{\mathrm{Absorbance}}_{\mathrm{sample}}\right)/{\mathrm{Absorbance}}_{\mathrm{control}}\right)]\times 100$$

#### 5.5.2 Inhibition of Albumin Denaturation Assay

The anti-inflammatory property of the SEDDS containing *H. carinata* extract was investigated using a modified albumin denaturation assay.^58^ The assay mixture contained 25 μL of egg albumin (fresh hen’s egg), 350 μL of PBS (0.1 M, pH 7.2), and 250 μL of either the test sample or PBS (control). The mixtures were incubated at 37 °C for 15 min and then heated at 70 °C for 5 min to induce protein denaturation. Following cooling on ice for 5 min, the absorbance was measured at 660 nm. Diclofenac sodium (Merck Ltd., Bangkok, Thailand) was used as the reference standard, with concentrations of 0, 50, 100, 250, and 500 μg/mL prepared under identical conditions. Results were expressed as diclofenac sodium equivalents (μg/mL). The percentage inhibition of albumin denaturation was calculated using the formula and reported as IC_50_ values:

$$\mathrm{Inhibition of albumin denaturation}\left(\%\right)=\left[1-\left({\mathrm{Absorbance}}_{\mathrm{sample}}/{\mathrm{Absorbance}}_{\mathrm{control}}\right)\right]\times 100$$

### 5.6 Animal Handling and Ethical Approval

Young adult male C57BL/6NJcl mice (3 months old) were used. The sample size was determined using G*Power software, version 3.1.9.6.^59^ Animals were procured from Nomura Co. Ltd. (Bangkok, Thailand) and maintained at the Animal Unit, Walailak University, under controlled conditions (25 ± 2 °C, 40–50% humidity, 12 h light/dark cycle) with free access to standard food and water. Mice were randomly assigned (four per cage), and oral treatments were administered daily between 07:00–08:00 A.M. All procedures were approved by the Institutional Animal Experiment Committee of Walailak University (Approval No. WU-ACUC-67056) and conducted in accordance with institutional and national guidelines for animal care.

### 5.7 Experimental Procedure

Eighty eight male C57BL/6NJcl mice (3 months old, 25–35 g) were randomly assigned into ten groups (*n* = 8 per group), and treatments were administered daily via oral gavage for 28 days. Group I served as the normal control and received no treatment. Group II received intraperitoneal MPTP^60^ (20 mg/kg BW × 4 doses at 2 h intervals on day 21) and deionized water as a vehicle. Group III received MPTP and the blank SEDDS. Group IV received MPTP and the reference drug Stalevo (25 mg/kg BW, a positive control drug for motor function). Group V received MPTP and vitamin C (100 mg/kg BW) as a positive control substance for antioxidant. Groups VI–VIII were treated with *H. carinata* extract at 0.50, 0.75, and 1.00 μmol/kg BW, respectively, in combination with MPTP. Groups IX–XI received SEDDS formulations of *H. carinata* extract at doses of 0.50, 0.75, and 1.00 μmol/kg BW, respectively, following MPTP administration.

### 5.8 Behavioral Analyses

Motor coordination and Parkinsonian-like symptoms were evaluated using the grid walk, narrow beam, and resting tremor rating scales prior to and on days 1, 3, and 7 following MPTP administration.

#### 5.8.1 Resting Tremor

Mice were evaluated for Parkinsonian-like resting tremors. Each mouse was placed in a square observation box elevated 500 cm above the floor and recorded on video for 45 min. The severity of resting tremor was scored according to the following criteria: 0 = no resting tremor observed; 1 = slight tremor in postural muscles; 2 = moderate tremor occasionally involving the head; 3 = marked tremor, not always affecting the head; 4 = continuous tremor without head or limb movement; and 5 = continuous tremor affecting the entire body.^61^

#### 5.8.2 Grid Walk

The grid-walking test was employed to assess motor impairment, specifically foot faults and deficits in paw placement accuracy during locomotion. Mice were placed on a flat metal grid (40 cm wide × 60 cm long) suspended 100 cm above the floor. In healthy mice, paw placement is normally precise to support body weight during movement across the grid. Each instance in which a paw slipped through an opening in the grid was recorded as a foot fault.^62^

#### 5.8.3 Narrow Beam

To evaluate balance, coordination, and fine motor skills, mice were trained to traverse a narrow wooden beam (100 cm in length × 1 cm in width) positioned 100 cm above the floor, leading to a sheltered escape platform. The latency to reach the platform was recorded as a measure of motor performance.^63^

Following behavioral tests, mice were euthanized with pentobarbital-HCl (50 mg/kg body weight, *i.p.*). Brains were dissected, with one hemisphere used for biochemical assays (glutathione peroxidase activity and malondialdehyde and nitrite levels) and the other for qPCR analysis of TNF-α and TH, as well as immunohistochemistry of TH.

### 5.9 The Antioxidant and Antineuroinflammatory Activities of SEDDS Containing *H. carinata* Extract in Mouse Model of Parkinsonian-Like Symptoms

#### 5.9.1 Assay of Lipid Peroxidation

Lipid peroxidation was assessed by measuring malondialdehyde (MDA) levels using the method.^64^ Brain samples were homogenized in ice-cold 20 mM PBS (pH 7.4) containing 0.5 mM butylated hydroxytoluene, then centrifuged at 3,000 × *g* for 10 min at 4 °C. A 200 μL aliquot of the supernatant was used to quantify MDA and 4-hydroxyalkenal (HAE) according to the manufacturer’s protocol. Results were normalized to protein content using a BCA assay kit.

#### 5.9.2 Assessment of Nitrite Levels

Brain tissue nitrite levels were quantified by a modified fluorometric method.^65^ In brief, 0.5 mL of 0.04% 4-hydroxycoumarin was added to 1 mL of supernatant, vortexed, and placed on ice for 5 min. The mixture was then supplemented with 50 μL of 8% sodium thiosulfate and incubated at 25 °C for 5 min, followed by the addition of 0.5 mL of 1.5 M NaOH. Fluorescence was measured using a spectrofluorometer FP-8200 (Jasco, UK) at 347 nm excitation and 453 nm emission, and values were expressed as nmol/mg protein.

### 5.10 qPCR Analysis of TH and TNF-α

Total RNA was extracted from striatum and cortex tissues using TRI Reagent (Molecular Research Center, USA). Complementary DNA (cDNA) was synthesized from 1 μg of total RNA with the ReverTra Ace qPCR RT Master Mix with gDNA Remover (TOYOBO, Japan) on a T100 Thermal Cycler (Bio-Rad, United States). qPCR was conducted in duplicate using Thunderbird Next SYBR qPCR Mix (TOYOBO, Japan) on a QuantStudio 6 Flex Real-Time PCR System (Applied Biosystems, United States). The thermal cycling protocol consisted of an initial denaturation at 95 °C for 10 min, followed by 45 cycles of denaturation at 95 °C for 15 s and annealing/extension at gene-specific temperatures (GAPDH: 56.7 °C; TH: 58.4 °C; TNF-α: 59.6 °C). A melt curve analysis (95 °C for 15 s, 60 °C for 60 s, and 95 °C for 15 s) was performed to verify amplification specificity. Relative mRNA expression levels of TH and TNF-α were normalized to the endogenous control GAPDH and calculated using the 2^–ΔΔCt^ method.^66^ Primer sequences were designed and synthesized by Macrogen (Seoul, South Korea), as presented in Table 7.

**Table 7:** Primer Sequences Used for qPCR

Genes	Primer Sequences	Reference
GAPDH	Forward: 5′-ACC ACA GTC CAT GCC ATC AC-3′	^67^
	Reverse: 5′-TCC ACC ACC CTG TTG CTG TA-3′	
TH	Forward: 5′-AAG CTG ATT GCA GAG ATT GCC-3′	^68^
	Reverse: 5′-TTC CTC CTT TGT GTA TTC CAC GT-3′	
TNF-α	Forward: 5′-TCT CAT CAG TTC TAT GGC CC-3′	^69^
	Reverse: 5′-GGG AGT AGA CAA GGT ACA AC-3′	

### 5.11 Immunohistochemistry for TH

Brain tissues were fixed in 4% paraformaldehyde overnight at 4 °C, cryoprotected in 30% sucrose, and embedded in OCT compound. Coronal paraffin sections (10 μm) were prepared to include the substantia nigra (bregma −2.70 mm). Sections were processed using a mouse anti-TH monoclonal antibody (1:100) and visualized with a DAB detection kit (Thermo Fisher Scientific Inc., Waltham, United States). Endogenous peroxidase activity was blocked with 10% methanol/0.3% H_2_O_2_, followed by blocking with 3% normal goat serum. After incubation with the primary antibody overnight at 4 °C, sections were treated with HRP-conjugated secondary antibody (1:500) for 2 h. Negative controls omitted the primary antibody. Slides were counterstained with hematoxylin, dehydrated, and mounted. TH-positive cells were imaged at 200× magnification using a microscope (OLYMPUS-BX43, Japan).

### 5.12 Statistical Analysis

Data are expressed as mean ± standard deviation (SD). One-way ANOVA followed by Tukey’s post hoc test was used to assess statistical differences among groups. A *p*-value of less than 0.05 was considered statistically significant. All graphs were conducted using GraphPad Prism software version 10.6.1, and statistical analyses were determined using SPSS software version 29.0.1.0.

## Supplementary Material



## Data Availability

All data required to substantiate the conclusions of this study are provided in the main text and/or the Supporting Information. Further data relevant to this work may be obtained from the authors.

## Notes

Extracted ion chromatogram (EIC) of Huperzine A and MS/MS fragmentation (Figure S1); EIC of Huperzine B and MS/MS fragmentation (Figure S2); EIC of des-*N*-methyl-β-obscurine and MS/MS fragmentation (Figure S3); EIC of Lycopodine and MS/MS fragmentation (Figure S4); EIC of Lycodine and MS/MS fragmentation (Figure S5); EIC of Fawcettimine and MS/MS fragmentation (Figure S6); EIC of Huperzine C and MS/MS fragmentation (Figure S7); EIC of α-obscurine and MS/MS fragmentation (Figure S8); EIC of β-obscurine and MS/MS fragmentation (Figure S9); EIC of Huperzinine and MS/MS fragmentation (Figure S10); EIC of Lycopodine-*N*-oxide and MS/MS fragmentation (Figure S11) (PDF)

## Abbreviations

HupA: Huperzine A
*H. carinata*: *Huperzia carinata* (Desv. ex Poir.) Trevis
SEDDS: Self-emulsifying drug delivery system
PD: Parkinson’s disease
SNpc: Substantia nigra pars compacta
ROS: Reactive oxygen species
MAO-B: Monoamine oxidase-B
COMT: Catechol-O-Methyltransferase
AChE: Acetylcholinesterase
O/W: Oil-in-water
AD: Alzheimer’s disease
BCS: Biopharmaceutics Classification System
SMEDDS: Self-microemulsifying drug delivery system
AUC: Area under the curve
C_max_: Maximum plasma concentration
LC-MS/MS: Liquid chromatography–tandem mass spectrometry
HPLC/ESI-QTOF: High-performance liquid chromatography coupled with electrospray ionization quadrupole time-of-flight mass spectrometry
PEG: Polyethylene glycol
PDI: Polydispersity index
TEM: Transmission electron microscopy
SGF: Simulated gastric fluid
SIF: Simulated intestinal fluid
DPPH: 2,2-diphenyl-1-picrylhydrazyl
MPTP: 1-methyl-4-phenyl-1,2,3,6-tetrahydropyridine
MDA: Malondialdehyde
GPx: Glutathione peroxidase
qPCR: Quantitative real-time polymerase chain reaction
IHC: Immunohistochemistry
TH: Tyrosine hydroxylase
TNF-α: Tumor necrosis factor-alpha
DA: Dopamine
GI tract: Gastrointestinal tract
NO: Nitric oxide
iNOS: Inducible nitric oxide synthase
IL: Interleukin
CD16: Cluster of differentiation 16;
HupB: Huperzine B
HAE: 4-Hydroxyalkenal
BCA assay: Bicinchoninic acid assay
PBS: Phosphate-buffered saline
IC50: Half-maximal inhibitory concentration
cDNA: Complementary DNA
GAPDH: Glyceraldehyde 3-phosphate dehydrogenase
OCT: Optimal cutting temperature
HRP: Horseradish peroxidase
