# Supplementary material for: Self-Emulsifying Formulation of Huperzia carinata Extract to Improve Huperzine A Solubility for Therapeutic Efficacy Enhancement in Parkinsonian Mice
Source: ACS Omega. 2026 Aug 5;11(33):49717–32. doi: 10.1021/acsomega.6c04075 (PMC13520518; doi:10.1021/acsomega.6c04075)
Supplement: Supplementary file 1 [file ao6c04075_si_001.pdf]

Supporting information

Self-Emulsifying Formulation of *Huperzia carinata* Extract to Improve Huperzine A Solubility for Therapeutic Efficacy Enhancement in Parkinsonian Mice

Namfa Sermkaew[1,2], Nuttapon Songnaka[1,2], Wuttipong Masraksa[3], Amit Jaisi[4], Yosita Yongyuen[1], Pannatorn Buntha[1], Nadiah Syafiqah Nor Azman[5], Juntratip Jomrit[1], and Phetcharat Boonruamkaew[1,*]

[1] School of Pharmacy, Walailak University, Nakhon Si Thammarat 80160, Thailand;

[2] Drug and Cosmetics Excellence Center, Walailak University, Nakhon Si Thammarat 80160, Thailand;

[3] Faculty of Medicine, Vongchavalitkul University, Nakhon Ratchasima, 30000, Thailand;

[4] Faculty of Science, Chulalongkorn University, Bangkok, 10330, Thailand;

[5] Faculty of Pharmacy, Lincoln University college, Selangor 47301, Malaysia;

[*]Correspondence author: phetcharat.bo@wu.ac.th or pairwaka@gmail.com

AUTHORS INFORMATION

Corresponding Author

[*]**Phetcharat Boonruamkaew** – *School of Pharmacy, Walailak University, Nakhon Si Thammarat 80160, Thailand*; E-mail**:** phetcharat.bo@wu.ac.th or pairwaka@gmail.com

Authors

Namfa Sermkaew– *School of Pharmacy, Walailak University, Nakhon Si Thammarat 80160, Thailand; Drug and Cosmetics Excellence Center, Walailak University, Nakhon Si Thammarat 80160, Thailand*

Nuttapon Songnaka– *School of Pharmacy, Walailak University, Nakhon Si Thammarat 80160, Thailand; Drug and Cosmetics Excellence Center, Walailak University, Nakhon Si Thammarat 80160, Thailand*

Wuttipong Masraksa – *Faculty of Medicine, Vongchavalitkul University, Nakhon Ratchasima, 30000, Thailand*

Amit Jaisi– *Faculty of Science, Chulalongkorn University, Bangkok, 10330, Thailand*

Yosita Yongyuen − *School of Pharmacy, Walailak University, Nakhon Si Thammarat 80160, Thailand*

Pannatorn Buntha − *School of Pharmacy, Walailak University, Nakhon Si Thammarat 80160, Thailand*

Nadiah Syafiqah Nor Azman – *Faculty of Pharmacy, Lincoln University College, Selangor 47301, Malaysia*

Juntratip Jomrit− *School of Pharmacy, Walailak University, Nakhon Si Thammarat 80160, Thailand*

Supplementary materials

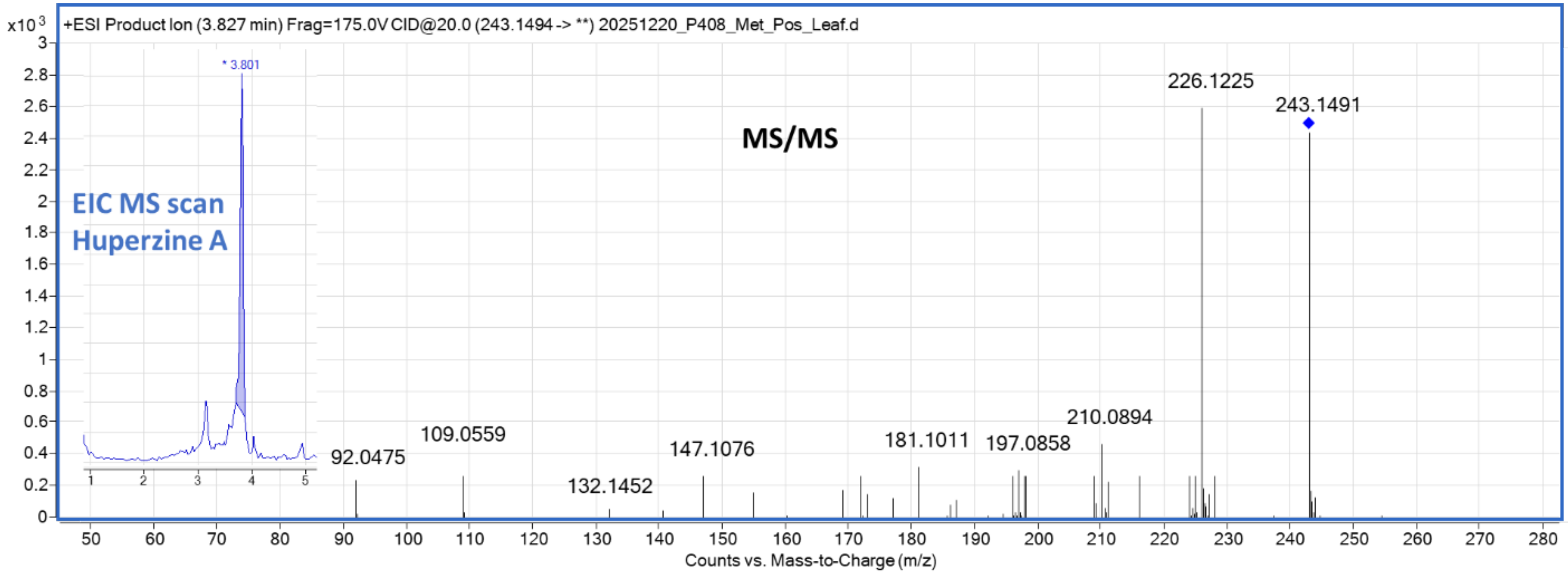

Figure S1. Extracted Ion Chromatogram (EIC) of Huperzine A and MS/MS Fragmentation

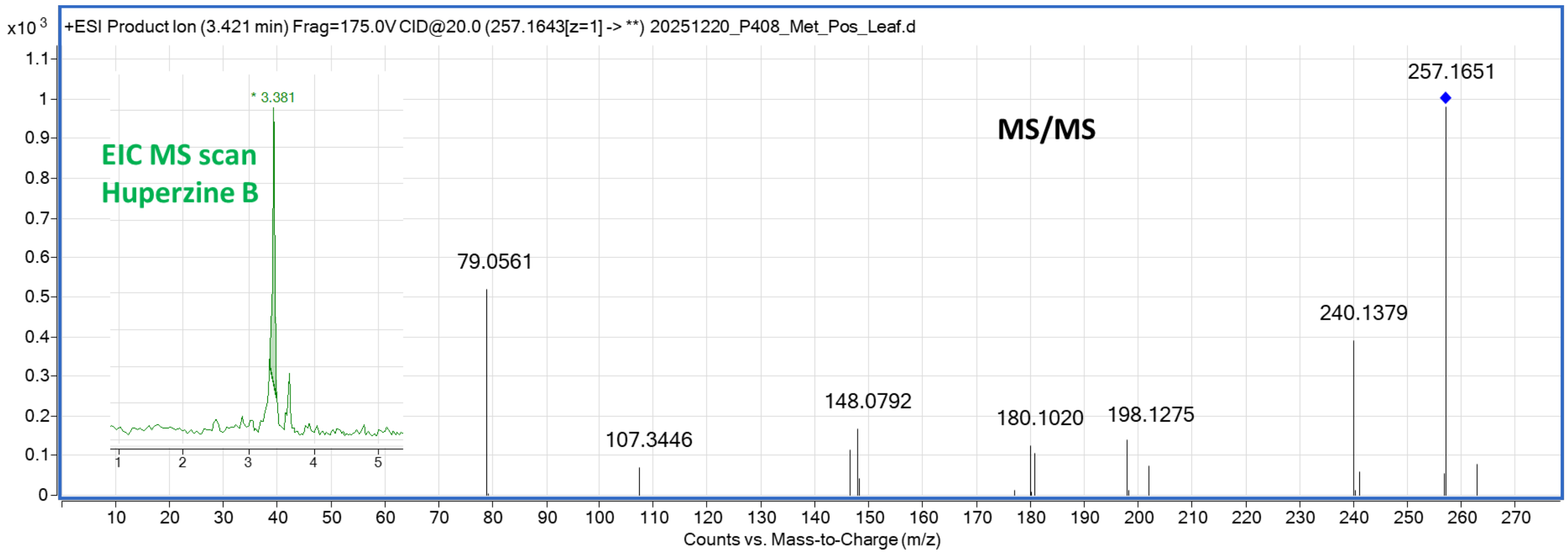

Figure S2. EIC of Huperzine B and MS/MS Fragmentation

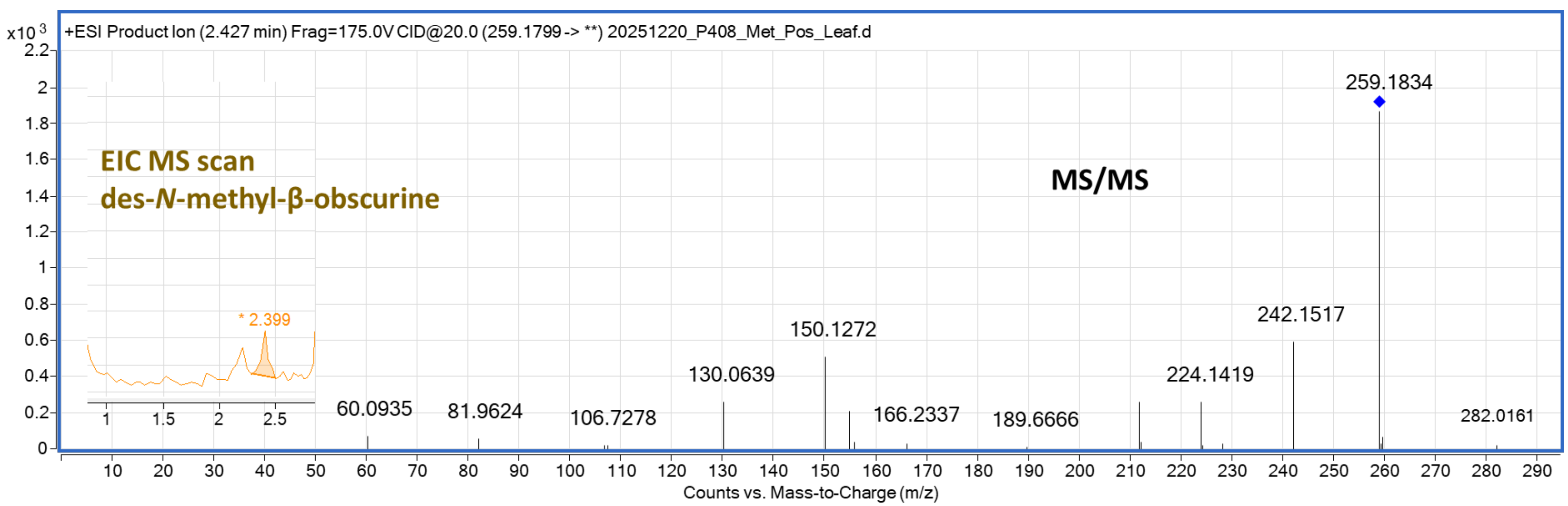

Figure S3. EIC of des-*N*-methyl-β-obscurine and MS/MS Fragmentation

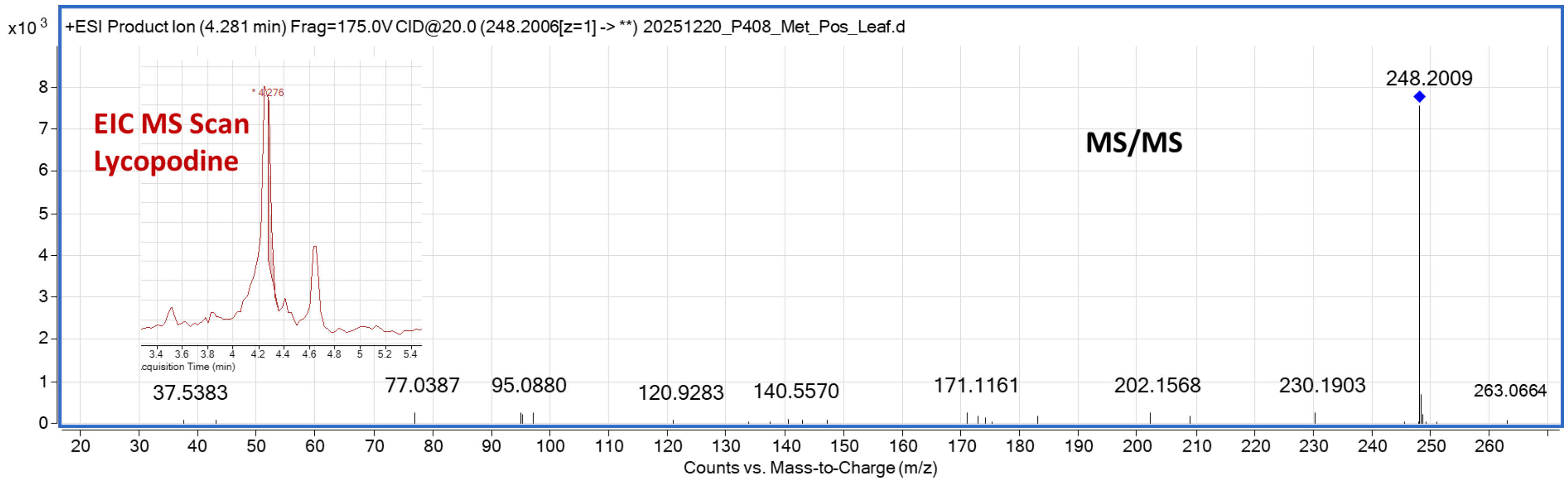

Figure S4. EIC of Lycopodine and MS/MS Fragmentation

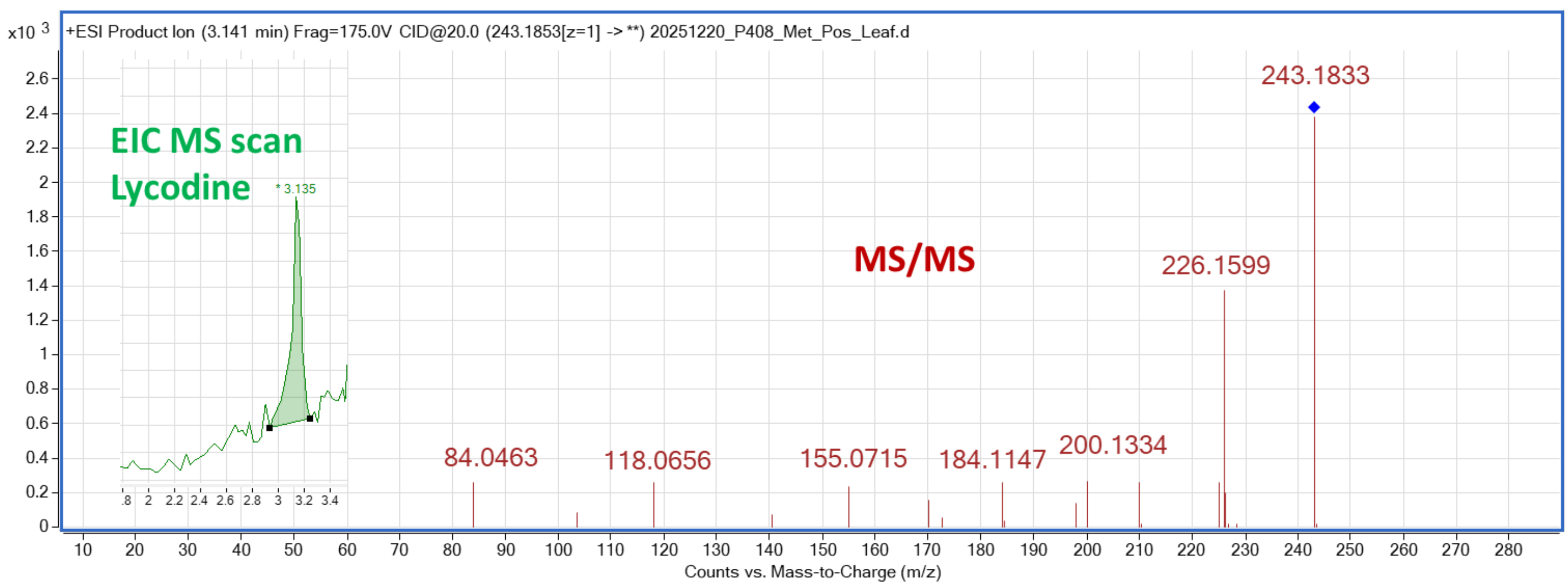

Figure S5. EIC of Lycodine and MS/MS fragmentation

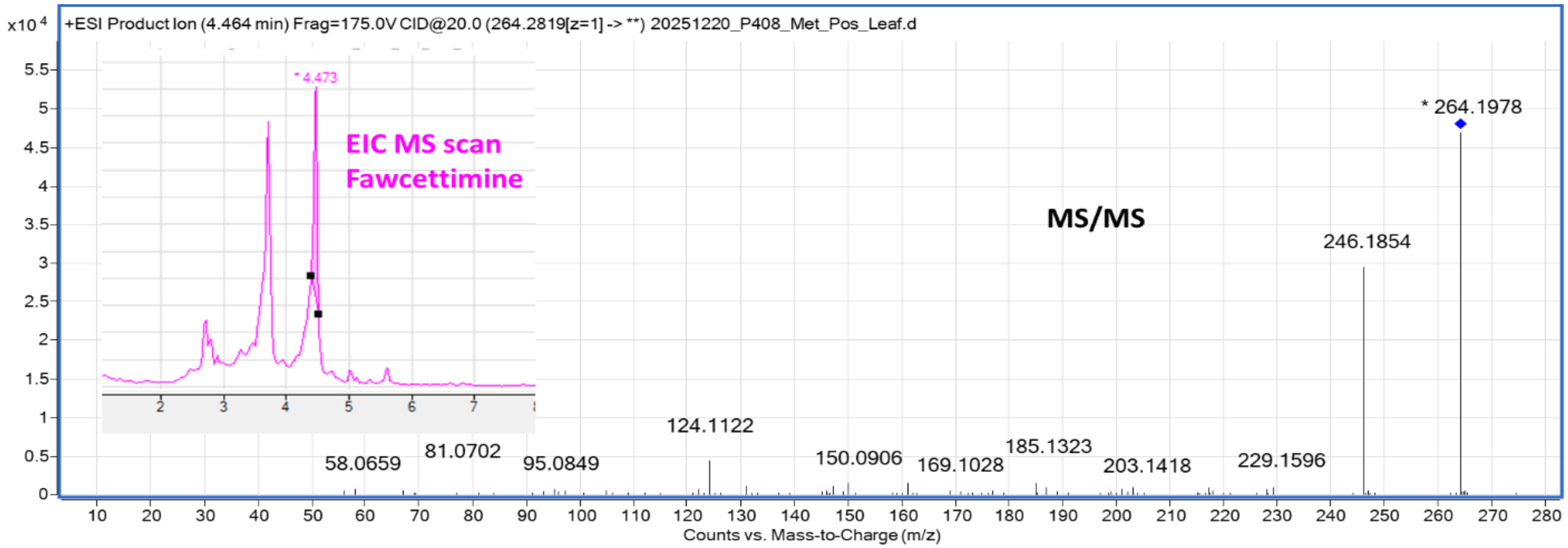

Figure S6. EIC of Fawcettimine and MS/MS fragmentation

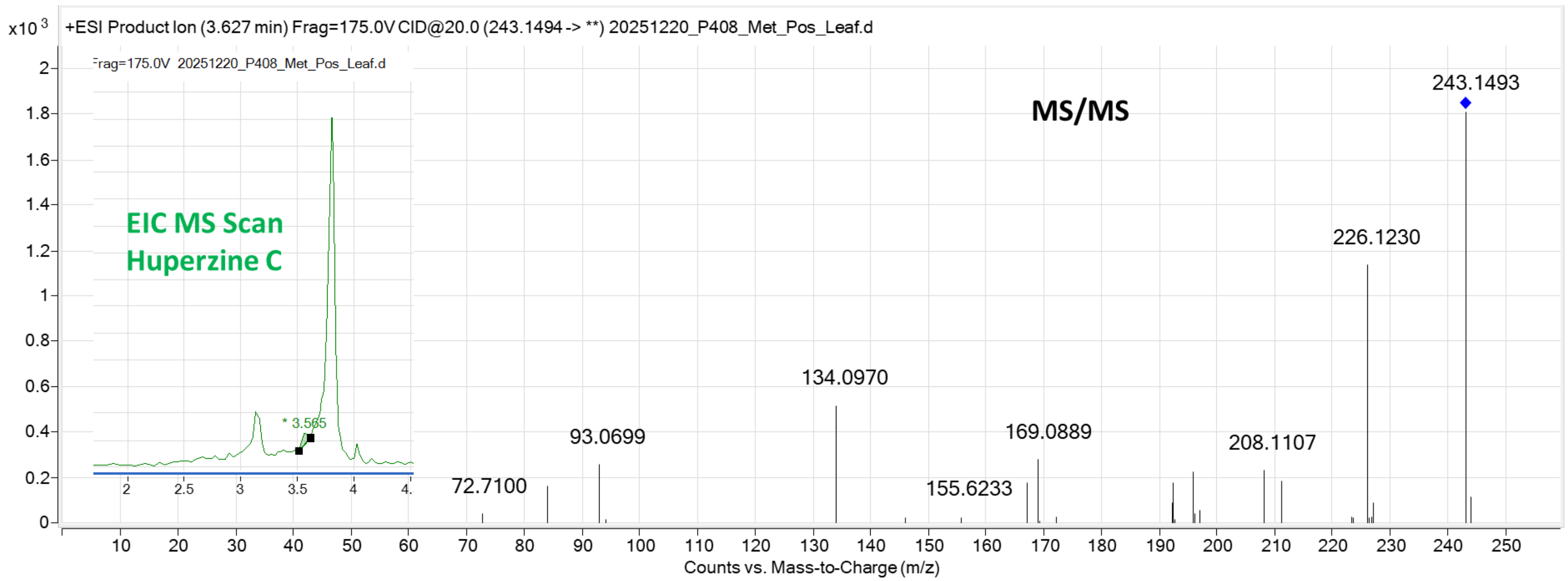

Figure S7. EIC of Huperzine C and MS/MS fragmentation

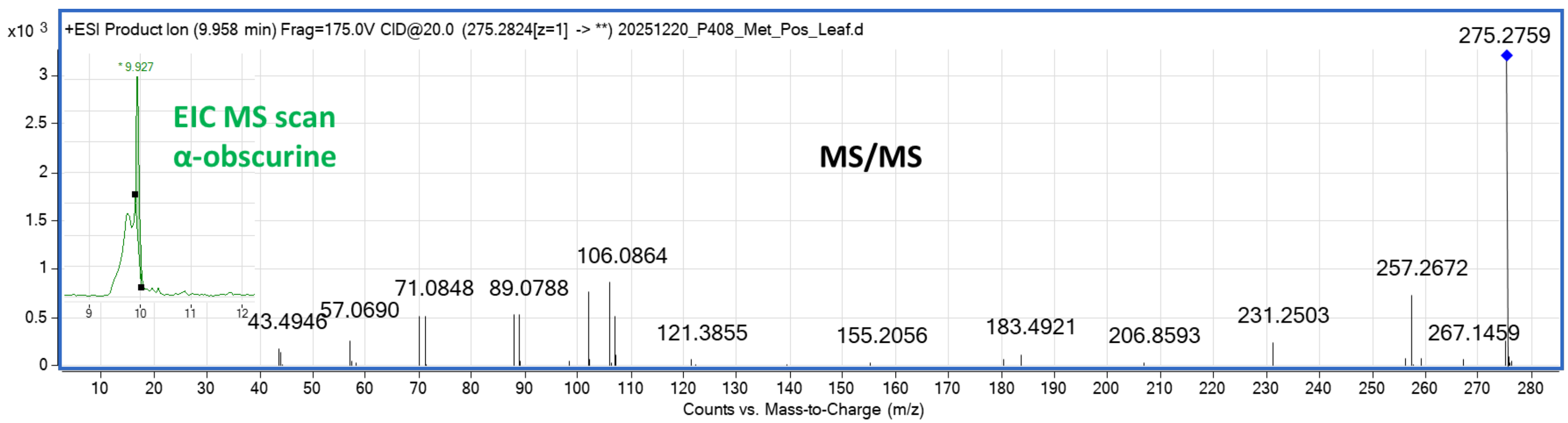

Figure S8. EIC of α-obscurine and MS/MS fragmentation

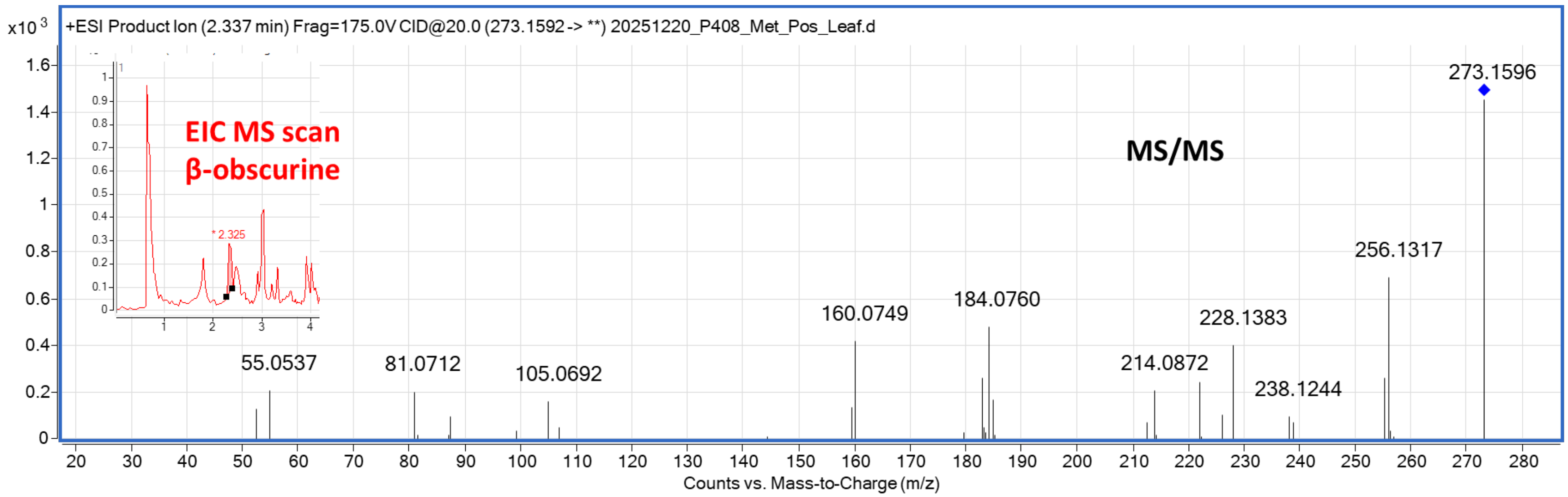

Figure S9. EIC of β-obscurine and MS/MS fragmentation

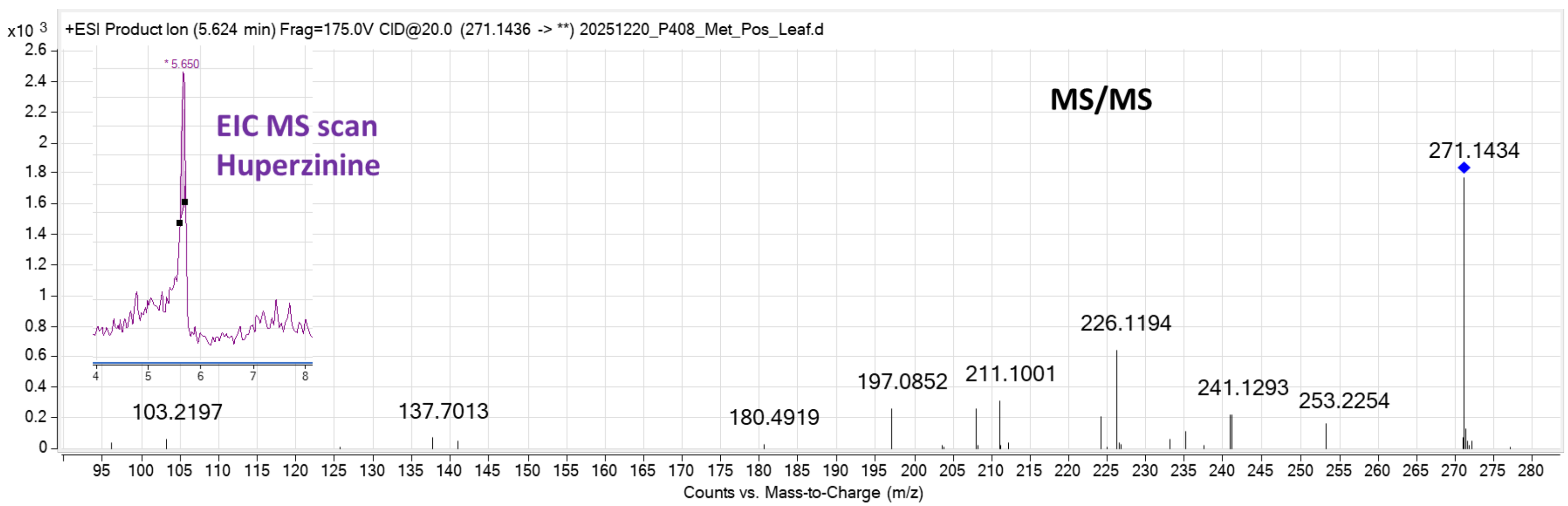

Figure S10. EIC of Huperzinine and MS/MS fragmentation

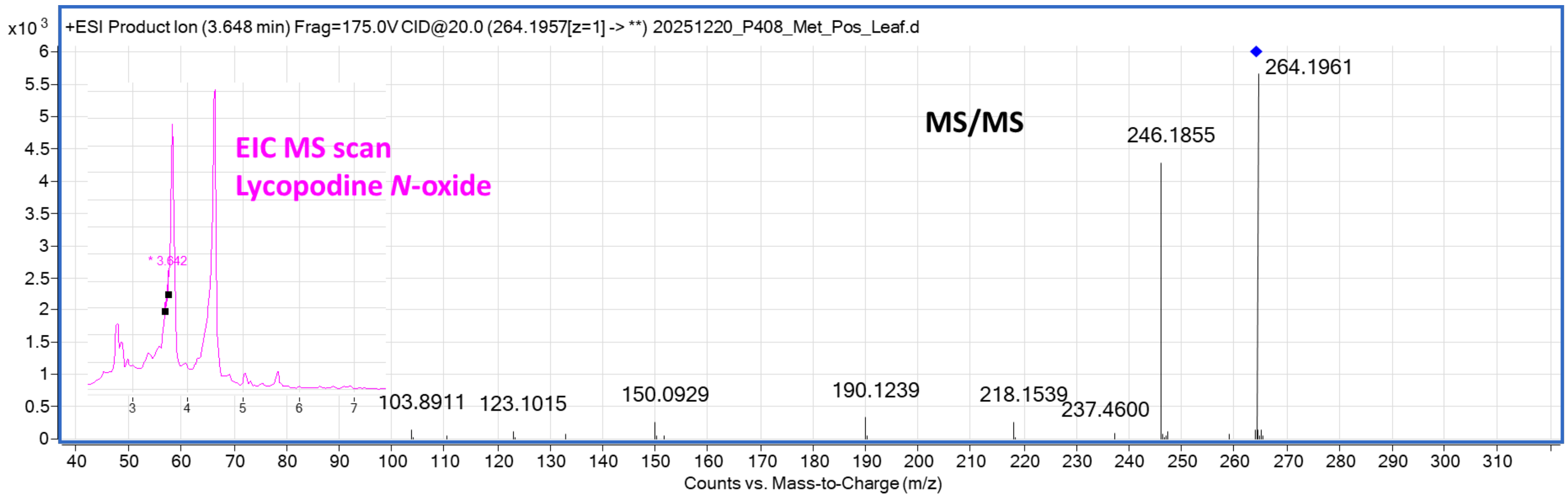

Figure S11. EIC of Lycopodine-*N*-oxide and MS/MS fragmentation
